# Medication-related osteonecrosis of the jaw in cancer and osteoporosis patients: a retrospective seven-year single-center Norwegian cohort study of clinical profiles and documented soft tissue healing

**DOI:** 10.2340/aos.v85.46983

**Published:** 2026-10-09

**Authors:** Safiyye Süslü, Håvard J. Haugen, Tormod B. Krüger, Janne Elin Reseland, Bente Brokstad Herlofson

**Affiliations:** a Department of Biomaterials, Institute of Clinical Dentistry, University of Oslo, Oslo, Norway; b Department of Oral Medicine and Oral Surgery, Institute of Clinical Dentistry, University of Oslo, Oslo, Norway

**Keywords:** Osteonecrosis of the jaw, antiresorptive agents, neoplasms, osteoporosis, tooth extraction, retrospective studies

## Abstract

**Objective:**

To compare systemic profiles, oral findings, and documented soft tissue healing between cancer and osteoporosis patients with medication-related osteonecrosis of the jaw (MRONJ).

**Material and methods:**

This retrospective single-center cohort included 105 cases treated in 2013–2019 (56 cancer; 49 osteoporosis). Healing at last follow-up was analyzed using adjusted logistic regression and Firth sensitivity analysis.

**Results:**

Of 102 cases with known final status, 68 had documented healing. Osteoporosis cases were older, predominantly female, and had longer antiresorptive exposure than cancer cases (76.7 ± 11.1 vs. 70.0 ± 8.5 years; 102.5 ± 77.8 vs. 34.6 ± 28.5 months; both *p* < 0.001). Stage II predominated and Stage 0 was uncommon (3/105, 2.9%). Previous tooth extraction at the MRONJ lesion site was more frequent in osteoporosis (95.9% vs. 71.4%; *p* < 0.001). Surgical treatment was associated with higher healing odds (adjusted odds ratio [OR]: 11.12, 95% confidence interval [CI]: 4.12 to 33.35; *p* < 0.001), whereas cancer indication was associated with lower odds (adjusted OR: 0.23, 95% CI: 0.07 to 0.68; *p* = 0.011).

**Conclusions:**

Systemic profiles differed; oral patterns were similar. Non-random treatment and missing healing dates limit interpretation. Previous tooth extraction was the most frequent antecedent event; its indication and timing were unverified.

## Introduction

Medication-related osteonecrosis of the jaw (MRONJ) is the current umbrella term for jaw osteonecrosis associated with antiresorptive and other relevant bone-modifying or antiangiogenic therapies. Historically, the entity was first described as bisphosphonate-related osteonecrosis of the jaw (BRONJ) in early American Association of Oral and Maxillofacial Surgeons (AAOMS) position papers, before the terminology broadened to MRONJ as denosumab and other non-bisphosphonate agents entered routine use [1–4]. Because the present cohort spans that terminology transition, we use MRONJ consistently in this paper and refer to BRONJ only when discussing historical definitions.

Antiresorptive therapy is used across a wider clinical spectrum than osteoporosis alone. Lower-dose regimens are used for osteoporosis and other metabolic bone disorders, and bone-targeted treatment is also used for reducing the risk of cancer recurrence and for cancer treatment-induced bone loss in selected patients receiving endocrine or other anticancer therapy [2, 5–7]. Higher-intensity regimens are most commonly used in malignant diseases with skeletal involvement, but the distinction between “low-dose” and “high-dose” therapy is better understood as a continuum of treatment intensity rather than an absolute disease-based separation [8, 9].

Current pathobiology models emphasize interactions between suppressed bone remodeling, altered oral barrier repair, local infection/inflammation, and treatment-related changes in host response, rather than a single isolated mechanism [10–13]. Consistent with that framework, periodontal or periapical infection and dental extractions are repeatedly reported in MRONJ case series, but extraction often occurs in already diseased teeth, making causal interpretation difficult [14–16].

MRONJ management remains controversial. CContemporary position papers and consensus documents recognize both non-surgical and surgical approaches to MRONJ management, but comparisons of their outcomes remain vulnerable to confounding by indication [6, 7, 17, 18]. Cohort studies and systematic reviews report favorable outcomes with appropriately planned surgery [19–22]. Structured non-surgical wound care may also resolve MRONJ and remains an option for patients unable to undergo surgery [23], although other cohorts have reported limited healing and progression during non-surgical management [24]. Seluki et al.’s systematic review supports consideration of disease stage when selecting treatment [19]; contemporary position papers also emphasize the patient’s systemic condition and local disease extent [6, 7]. Prospective studies have additionally assessed quality of life following MRONJ treatment [25, 26].

Published studies address different aspects of MRONJ, including pharmacological management followed by removal of bone sequestra [27], MRONJ after tooth extraction in patients treated for osteoporosis [28], surgical outcomes [20], and survival among patients with cancer [29]. These studies differ in populations, treatment protocols, and outcome definitions, limiting direct comparison across indications. Consequently, well-characterized MRONJ cohorts are needed with comprehensive systemic and local data, clearly defined clinical outcomes, transparent follow-up reporting, and appropriately adjusted analyses.

This retrospective single-center Norwegian cohort study aimed to compare systemic profiles, oral findings, AAOMS stage distribution, recorded antecedent dental events, treatment patterns, follow-up characteristics, and documented clinical soft tissue healing status at the last recorded follow-up between cancer-related and osteoporosis-related MRONJ cases.

## Materials and methods

### Study design and patient selection

This retrospective single-center case-only cohort study was conducted at the Department of Oral Surgery and Oral Medicine, University of Oslo, Norway. All patients referred between July 2013 and December 2019 with a verified MRONJ diagnosis according to the contemporaneous AAOMS criteria were included in the analytical cohort; no patients with diagnosed MRONJ were excluded. All included patients consented to their inclusion in the study and the registration of their clinical data for research. The cohort therefore comprised 105 consecutive MRONJ cases, including 56 cancer-related and 49 osteoporosis-related cases. The study was approved by the Regional Committee for Medical and Health Research Ethics South-East B, Norway (REK 2013/1053).

### Case definition and terminology

Cases were diagnosed during the study period according to contemporaneous AAOMS criteria. For this manuscript, all cases are described as MRONJ, with historical BRONJ terminology retained only when discussing earlier AAOMS position papers [1, 2]. The 2022 AAOMS position paper [7] informed the terminology and conceptual framing of this revision, but cases were not retrospectively re-adjudicated beyond harmonized staging. Staging was harmonized as Stage 0, I, II, or III; non-exposed disease in the dataset was treated as Stage 0. Because Stage 0 is diagnostically non-specific and no structured radiographic dataset was available for systematic retrospective re-evaluation, Stage 0 analyses were interpreted descriptively and examined in sensitivity analyses.

### Exposure classification

The primary clinical grouping was cancer-related versus osteoporosis-related MRONJ, based on the recorded indication for bone-modifying agent (BMA) treatment. A proxy high-dose versus low-dose BMA classification could be derived by combining indication, agent, route, and dosing frequency. Exact administered dose, cumulative exposure, adherence, and detailed switching chronology were not available in a sufficiently complete form. In this dataset, the proxy was concordant with the indication grouping and therefore provided no independent information; it was retained only as a transparency check and was not included in the same adjusted model as the indication (Supplementary Table S11).

### Variables and data collection

The dataset included age, sex, indication for antiresorptive therapy, cancer subtype where applicable, antiresorptive agent, route, dosing frequency, and antiresorptive therapy (ART) duration. Oral variables included MRONJ stage, jaw location, lesion-site dental findings, tooth attrition/bruxism, a recorded history of previous tooth extraction at the MRONJ lesion site, denture trauma, and prior endodontic treatment. Pain (visual analogue scale [VAS]) was recorded but was not retained in the final comparative table because the coding range was inconsistent. Among the 87 patients with an extraction recorded at the MRONJ lesion site, tooth mobility, local endodontic treatment, tooth or crown fracture, previous deep caries, apical periodontitis, and generalized marginal bone loss were recorded as possible clinical reasons or dental conditions associated with extraction. However, these non-mutually-exclusive variables were not labelled in the dataset as verified extraction indications, and their timing relative to extraction and MRONJ recognition was unavailable. Implant involvement was recorded separately as local implant-related context (Figure 1; Supplementary Table S8).

**Figure 1:**
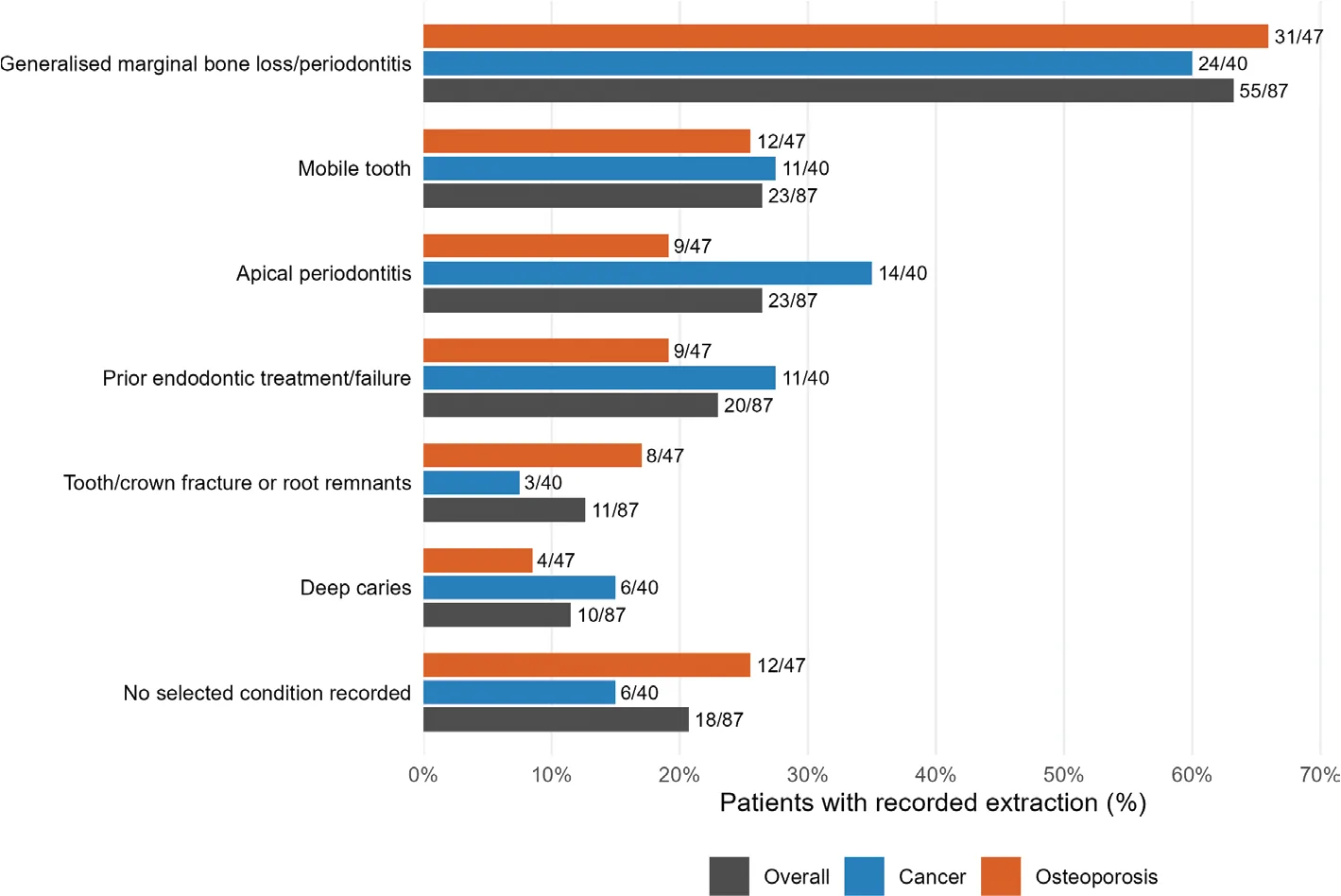
Recorded possible reasons or local dental conditions associated with extraction among patients with an extraction recorded at the MRONJ lesion site. Bars show the proportions of multiple responses overall (*n* = 87), in cancer-related cases (*n* = 40), and in osteoporosis-related cases (*n* = 47). Individual patients could have more than one recorded condition, so percentages do not sum to 100%.

Systemic variables included predefined comorbidities and comedications recorded at the time of MRONJ diagnosis. However, a valid full Charlson derivation was not possible because several required diagnostic categories were absent, aggregated, or insufficiently specific in the dataset.

### Outcomes, treatment definitions, and follow-up

Treatment was classified into two groups: non-surgical management and surgical treatment. Surgical treatment included sequestrectomy, debridement, or marginal bone resection followed by primary soft tissue closure using local flaps. This patient cohort underwent no segmental resections. According to the clinic’s general treatment protocol, antibiotics and antimicrobial mouth rinses formed part of surgical care and were used when clinically indicated during non-surgical management. Patient-level regimen, duration, and local wound-care data were not available in a sufficiently structured form for analysis. All included patients discontinued ART after their MRONJ diagnosis, at the discretion of the treating oncologist or osteologist. This represented post-diagnosis ART discontinuation. Bedogni et al. distinguish this therapeutic context from a prophylactic drug holiday before dentoalveolar surgery in patients without established or suspected MRONJ [6]. Exact timing and duration were unavailable, and the independent effect of post-diagnosis discontinuation could not be evaluated.

Clinical soft tissue healing was defined as documented mucosal coverage in the area of previously exposed bone or a final recorded status of soft tissue healed at the affected site. The structured dataset captured the follow-up duration, in months, from the start of MRONJ treatment to the last recorded follow-up visit. In contrast, the exact date of the first documented healing was unavailable. Accordingly, the primary analysis evaluated documented clinical soft tissue healing status at the last recorded follow-up rather than a conventional time-to-first-healing endpoint. No predefined minimum follow-up threshold was applied; the primary final-status analysis was restricted to cases with a known final clinical outcome. Mortality was summarized descriptively because the timing of death was recorded relative to MRONJ diagnosis rather than treatment start.

Panoramic radiography was routinely used, and cone-beam computed tomography (CBCT) was obtained for MRONJ diagnosis and thereafter when clinically indicated. Serial radiographic findings were not available in a standardized analytical dataset. Consequently, all reported healing outcomes refer to documented clinical soft tissue status and do not establish radiographic healing or complete disease resolution.

### Statistical methods

Group comparisons used Student’s t-test or Wilcoxon rank-sum test for continuous variables. Categorical variables were compared using Pearson’s chi-square test or Fisher’s exact test, as appropriate. *P*-values are reported to three decimal places, with *p* < 0.001 used for values below 0.001. To characterize differences between cancer-related and osteoporosis-related MRONJ within this case-only cohort, we first used descriptive comparisons. Then we fitted a multivariable logistic regression model with indication group (cancer vs. osteoporosis) as the dependent variable. Covariates were selected a priori from clinically relevant demographic, treatment, systemic, and oral variables. Model discrimination was assessed by the area under the receiver operating characteristic curve (AUC), and calibration was assessed by comparing observed and predicted probabilities across deciles of fitted probability. Because some variables showed quasi-complete separation, a Firth-penalized logistic regression was fitted as a sensitivity analysis. A reduced-sensitivity model, excluding chemotherapy and antiangiogenic therapy, assessed whether the contrast between indication groups persisted without explicitly including diagnosis-driven oncological treatment variables. Multiple correspondence analysis followed by k-means clustering was used as an exploratory descriptive visualization; because indication was included among the input variables, it was not interpreted as independent confirmation of indication-specific profiles.

Documented clinical soft tissue healing at the last recorded follow-up was analyzed as a binary endpoint. The primary multivariable model included treatment group, indication group, and baseline stage group (Stage 0/I, Stage II, Stage III). Standard logistic regression was fitted first, and Firth-penalized logistic regression was treated as a small-sample sensitivity analysis. Additional sensitivity analyses added sex, exploratory comedication burden, or both to the primary model; classified unknown final status as no documented healing; excluded Stage 0 cases; or added age or ART duration. A secondary model also accounted for the duration of documented clinical observation. The high-dose versus low-dose BMA proxy was concordant with indication and was therefore not treated as an independent sensitivity analysis. Because the number of non-healed outcomes was limited, no single model was interpreted causally or as definitive for all measured covariates; residual confounding by performance status and other unmeasured factors remains possible. Model diagnostics and analytic sample flow are provided in Supplementary Tables S9 and S10.

Exploratory comorbidity burden was examined using Poisson regression adjusted for age, with a second model additionally adjusted for sex. Follow-up duration was reported descriptively as months from treatment start to the last recorded follow-up visit. It was included only in secondary ascertainment analyses because it may be outcome-dependent. Exact binomial confidence intervals were used for key healed proportions. Supplementary Table S6 summarizes variable-level missingness.

## Results

### Study population and antiresorptive therapy characteristics

The cohort comprised 105 MRONJ cases: 56 cancer-related and 49 osteoporosis-related. Osteoporosis patients were older than cancer patients (76.7 ± 11.1 vs. 70.0 ± 8.5 years) and were more often female (43/49, 87.8% vs. 25/56, 44.6%; both *p* < 0.001). Antiresorptive exposure was markedly longer in osteoporosis than in cancer (102.5 ± 77.8 vs. 34.6 ± 28.5 months, *p* < 0.001), whereas cancer-related MRONJ clustered around monthly subcutaneous or intravenous regimens and osteoporosis-related MRONJ around oral weekly or other lower-frequency regimens (Table 1; Supplementary Figure S2).

**Table 1:** Baseline characteristics, antiresorptive therapy exposure, and systemic context by indication group.

Characteristic	Osteoporosis (*n* = 49)	Cancer (*n* = 56)	*P*
N	49	56	
Age, years	76.7 ± 11.1	70.0 ± 8.5	< 0.001
Sex: Male	6 (12.2%)	31 (55.4%)	< 0.001
Sex: Female	43 (87.8%)	25 (44.6%)	
ART duration, months	102.5 ± 77.8	34.6 ± 28.5	< 0.001
ART duration, years	7.0 [3.5, 11.0]	2.1 [1.5, 3.4]	< 0.001
Route: Oral	32 (65.3%)	0 (0.0%)	< 0.001
Route: Subcutaneous	7 (14.3%)	31 (55.4%)	
Route: Intravenous	10 (20.4%)	25 (44.6%)	
Dosing frequency: Weekly	33 (67.3%)	1 (1.8%)	< 0.001
Dosing frequency: Monthly	1 (2.0%)	46 (82.1%)	
Dosing frequency: Every 3 months	2 (4.1%)	1 (1.8%)	
Dosing frequency: Every 6 months	7 (14.3%)	5 (8.9%)	
Dosing frequency: Yearly	6 (12.2%)	0 (0.0%)	
Dosing frequency: Other	0 (0.0%)	3 (5.4%)	
Exploratory comorbidity score	2.0 ± 1.6	1.1 ± 1.1	0.002
Exploratory comedication score	1.9 ± 1.4	2.5 ± 1.1	0.012
Chemotherapy	3 (6.1%)	46 (82.1%)	< 0.001
Antiangiogenic therapy	0 (0.0%)	9 (16.1%)	0.003
Corticosteroids	18 (36.7%)	28 (50.0%)	0.172
Proton pump inhibitors	11 (22.4%)	13 (23.2%)	0.926

Continuous data are presented as mean ± SD or median (IQR), and categorical data as *n* (%). For multi-category variables, the overall *p*-value is shown once for the complete distribution. Categorical variables were compared using Pearson’s chi-square test or Fisher’s exact test, as appropriate; continuous variables were compared using Student’s t-test or the Wilcoxon rank-sum test.

SD: standard deviation; ART: antiresorptive therapy; IQR: interquartile range

The exploratory comorbidity score was higher in osteoporosis than in cancer (2.0 ± 1.6 vs. 1.1 ± 1.1, *p* = 0.002), whereas the exploratory comedication score was higher in cancer than in osteoporosis (2.5 ± 1.1 vs. 1.9 ± 1.4, *p* = 0.012) (Table 1). Supplementary Tables S2 and S3 provide detailed distributions of variable-level systemic comorbidity and concomitant medications.

In supplementary indication models, shorter antiresorptive treatment duration remained the clearest marker of cancer indication after exclusion of chemotherapy and antiangiogenic therapy, and the Firth-penalized sensitivity model yielded directionally concordant estimates (Supplementary Tables S4 and S5). Model discrimination and calibration were assessed in-sample and were good (Supplementary Figures S3 and S4). Multiple correspondence analysis provided a descriptive visualization of partially overlapping profiles; because indication was included in the analysis, it was not interpreted as independent confirmation of the two groups (Supplementary Figure S1).

### Oral findings, antecedent dental events, lesion distribution, and stage

A recorded history of previous tooth extraction at the MRONJ lesion site was present in 87/105 cases (82.9%) overall, including 47/49 osteoporosis cases (95.9%) and 40/56 cancer cases (71.4%; *p* < 0.001) (Table 2). Among these 87 extraction cases, the most frequently recorded possible reason or associated local dental condition was generalized marginal bone loss/periodontitis (55/87, 63.2%), followed by mobile teeth and apical periodontitis (both 23/87, 26.4%) and prior endodontic treatment/failure (20/87, 23.0%); individual patients could have more than one recorded condition. No selected condition was recorded in 18/87 cases (20.7%) (Figure 1; Supplementary Table S8; Supplementary Figure S7). Tooth attrition/bruxism was more frequent in cancer than in osteoporosis patients (19/56, 33.9% vs. 8/49, 16.3%; *p* = 0.040) (Table 2).

**Table 2:** Oral findings, recorded previous tooth extraction, and stage by indication group.

Characteristic	Osteoporosis (*n* = 49)	Cancer (*n* = 56)	*P*
MRONJ stage: Stage 0	2 (4.1%)	1 (1.8%)	0.380
MRONJ stage: Stage I	13 (26.5%)	12 (21.4%)	
MRONJ stage: Stage II	26 (53.1%)	26 (46.4%)	
MRONJ stage: Stage III	8 (16.3%)	17 (30.4%)	
Lesion site: Maxilla	14 (28.6%)	23 (41.1%)	0.208
Lesion site: Mandible	27 (55.1%)	29 (51.8%)	
Lesion site: Both jaws	8 (16.3%)	4 (7.1%)	
Previous tooth extraction at MRONJ lesion site	47 (95.9%)	40 (71.4%)	< 0.001
Mobile tooth at lesion site	13 (26.5%)	19 (33.9%)	0.411
Endodontic treatment/failure at lesion site	9 (18.8%)	16 (28.6%)	0.243
Tooth fracture at lesion site	9 (18.4%)	5 (8.9%)	0.156
Deep caries at lesion site	4 (8.5%)	6 (10.9%)	0.749
Apical periodontitis at lesion site	10 (20.8%)	19 (33.9%)	0.138
General marginal bone loss/periodontitis	32 (65.3%)	33 (58.9%)	0.502
Tooth attrition/bruxism	8 (16.3%)	19 (33.9%)	0.040

Overall *p*-values are shown once for the stage and lesion-site blocks. Categorical variables were compared using Pearson’s chi-square test or Fisher’s exact test, as appropriate. Percentages are based on available observations; variable-level missingness is summarized in Supplementary Table S6.

Lesion-site distribution was recorded as mandible-only in 56/105 cases (53.3%), maxilla-only in 37/105 cases (35.2%), and both jaws in 12/105 cases (11.4%). Posterior premolar/molar involvement was recorded in 93/105 cases (88.6%; 95% CI: 80.9–94.0%) (Figure 2). Individual patients could contribute more than one affected tooth site to the tooth-position plot.

**Figure 2:**
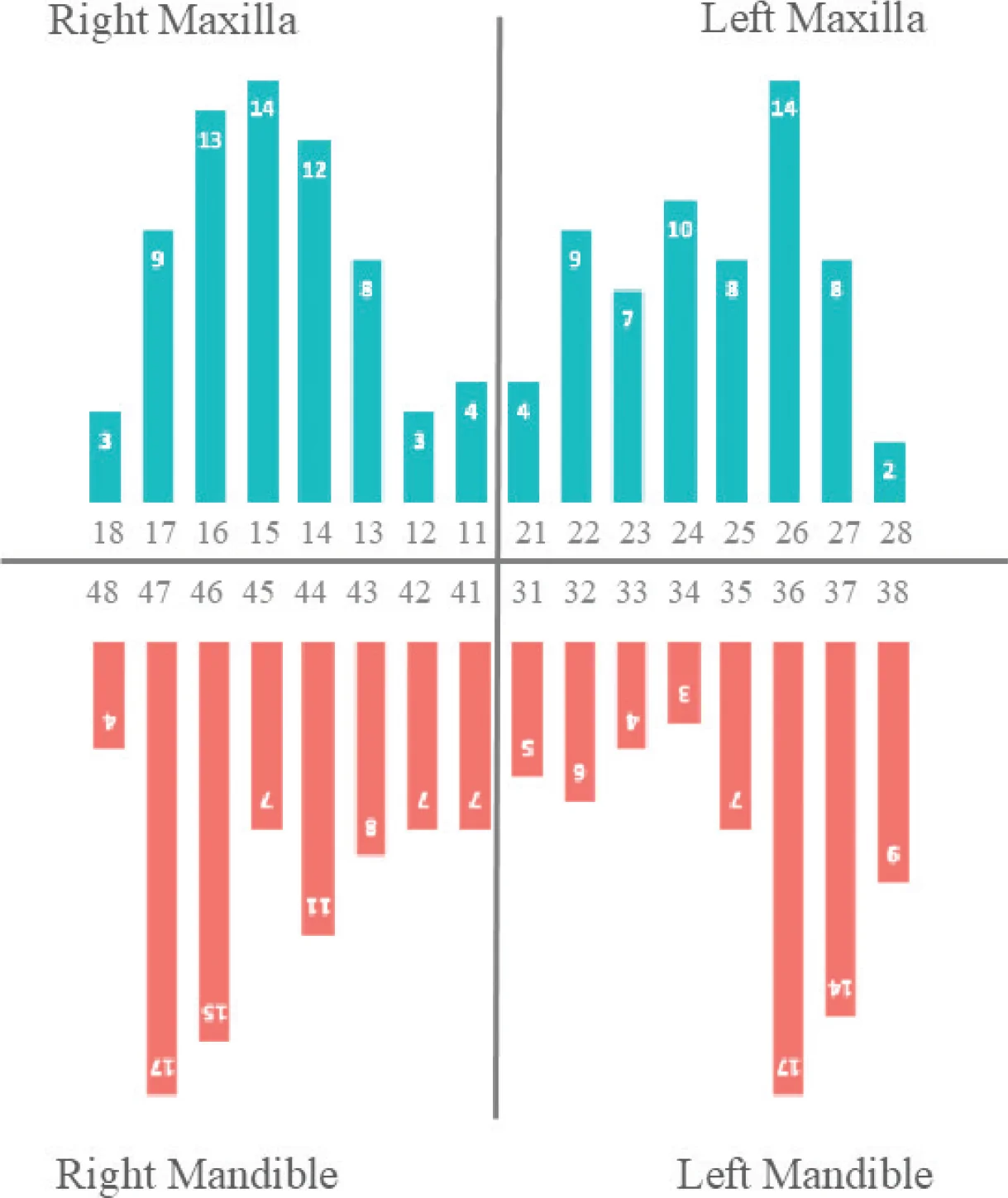
Dental sites of MRONJ lesions. Bars show affected tooth sites by region in the total study population (*n* = 105), separately for the mandible and maxilla; individual patients could contribute more than one affected tooth site. Patient-level exclusive jaw categories showed mandible-only involvement in 56/105 cases (53.3%), maxilla-only involvement in 37/105 cases (35.2%), and both-jaw involvement in 12/105 cases (11.4%).

Overall, Stage 0 occurred in 3/105 cases (2.9%), Stage I in 25/105 (23.8%), Stage II in 52/105 (49.5%), and Stage III in 25/105 cases (23.8%). Stage II predominated in both indication groups, and stage distributions did not materially differ between osteoporosis-related and cancer-related MRONJ (Figure 3; Table 2; Supplementary Table S7). Stage 0 or non-exposed disease was uncommon in both groups, accounting for 2/49 cases (4.1%) in osteoporosis and 1/56 cases (1.8%) in cancer. Supplementary Table S1 provides an expanded descriptive summary of stage, jaw localization, treatment, follow-up, and clinical outcome.

**Figure 3:**
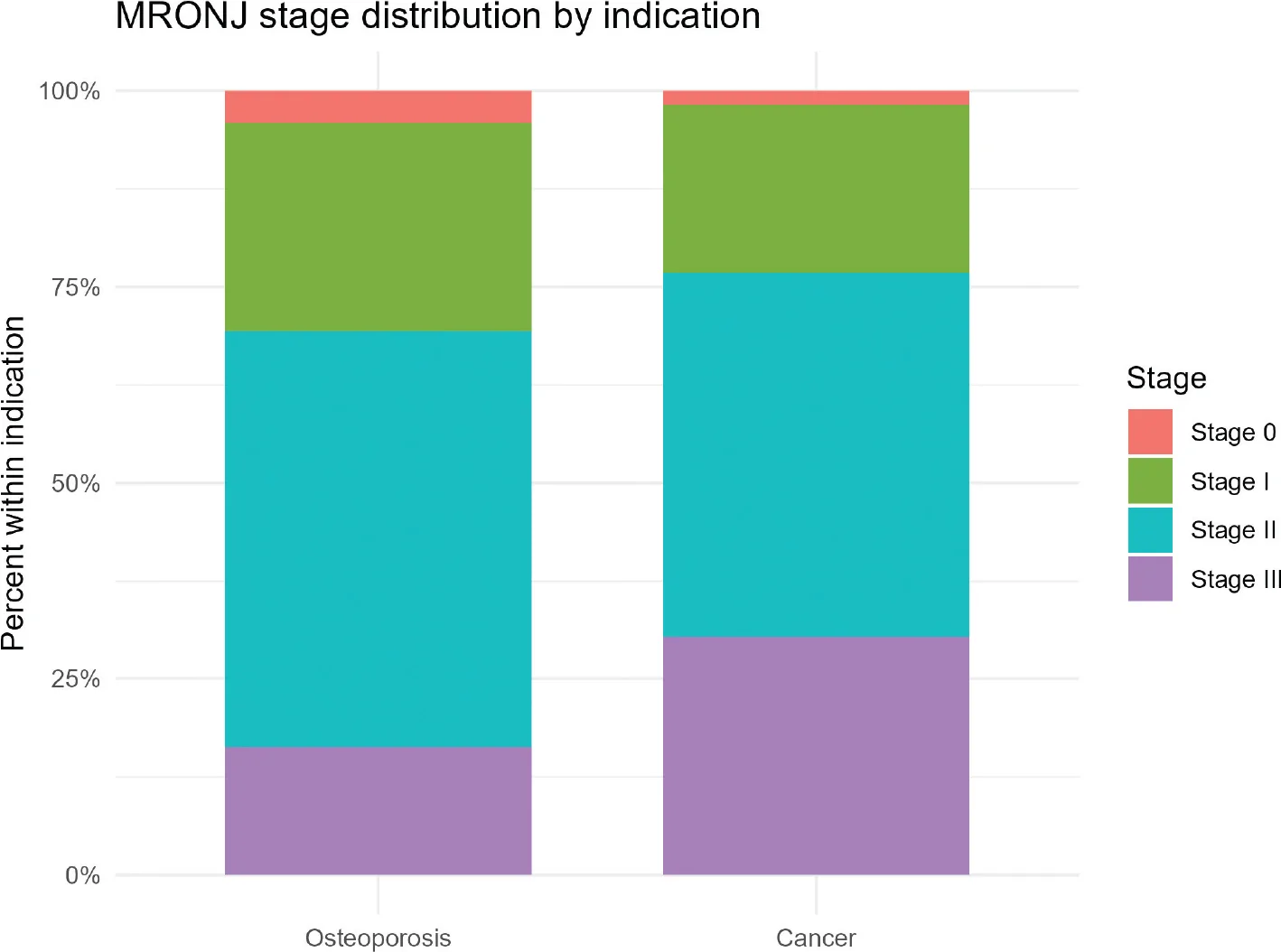
MRONJ stage distribution by indication group**.** Stacked bars show the percentage distribution of MRONJ stages within the osteoporosis and cancer groups. Stage II was the most frequent presentation in both groups, while Stage 0/non-exposed disease was uncommon. Cancer-related MRONJ showed a somewhat higher proportion of Stage III cases, whereas osteoporosis-related MRONJ showed a higher proportion of Stage I cases.

### Treatment, follow-up, and healing

Treatment distribution did not differ materially between indication groups (Table 3; Supplementary Table S1). Overall, 70/105 patients (66.7%) received surgical treatment and 35/105 (33.3%) received non-surgical management. Follow-up from treatment start was available for 103/105 cases, with a median of 10.0 months (interquartile range [IQR]: 5.5–17.0). No predefined minimum follow-up threshold was applied. Final clinical status was known for 102/105 cases; among these, documented clinical soft tissue healing was observed in 68/102 cases (66.7%), including 37/46 osteoporosis-related cases (80.4%) and 31/56 cancer-related cases (55.4%; *p* = 0.008) (Table 3). Among patients with a known final clinical outcome, documented soft tissue healing was recorded in 11/34 non-surgically managed patients (32.4%) and 57/68 surgically treated patients (83.8%; *p* < 0.001) (Supplementary Table S1B; Supplementary Figure S6). ART was discontinued in all patients after MRONJ diagnosis; exact timing and duration were not available. Death during follow-up was recorded more often in cancer than in osteoporosis (40/55, 72.7% vs. 15/46, 32.6%; *p* < 0.001) (Table 3).

**Table 3:** Treatment group, follow-up, and clinical outcomes by indication group.

Characteristic	Osteoporosis (*n* = 49)	Cancer (*n* = 56)	*P*
Treatment group: Non-surgical	14 (28.6%)	21 (37.5%)	0.333
Treatment group: Surgical	35 (71.4%)	35 (62.5%)	
Follow-up from treatment start, months	8.0 [5.0, 15.5]	11.0 [6.8, 17.5]	0.064
Final status available for healing analysis	46 (93.9%)	56 (100.0%)	0.098
Documented soft tissue healing among patients with known final status	37/46 (80.4%)	31/56 (55.4%)	0.008
Not healed among patients with known final status	9/46 (19.6%)	25/56 (44.6%)	
Death recorded during follow-up	15/46 (32.6%)	40/55 (72.7%)	< 0.001

The binary treatment-group comparison used Pearson’s chi-square test. Healing percentages use cases with known final status as the denominator. Follow-up is median (interquartile range [IQR]). Death status was available for 46 osteoporosis-related and 55 cancer-related cases.

When treatment groups were compared directly, patients receiving surgical treatment were more often female and had a lower exploratory comedication score than those receiving non-surgical management. Age, indication group, stage group, antiresorptive duration, exploratory comorbidity score, chemotherapy, and antiangiogenic therapy were similar on the measured baseline variables (Table 4). The exploratory treatment-selection model did not identify a dominant measured predictor, but residual confounding by clinical judgement, performance status, prognosis, and surgical suitability remains possible (Supplementary Table S12). Observed healing proportions by treatment group are shown in Supplementary Figure S6.

**Table 4:** Baseline characteristics by treatment group (non-surgical versus surgical treatment).

Characteristic	Non-surgical (*n* = 35)	Surgical (*n* = 70)	*P*
N	35	70	
Age, years	72.5 ± 10.7	73.4 ± 10.2	0.686
Sex: Male	18 (51.4%)	19 (27.1%)	0.014
Sex: Female	17 (48.6%)	51 (72.9%)	
Indication: Osteoporosis	14 (40.0%)	35 (50.0%)	0.333
Indication: Cancer	21 (60.0%)	35 (50.0%)	
Baseline stage group: Stage 0/I	9 (25.7%)	19 (27.1%)	0.446
Baseline stage group: Stage II	20 (57.1%)	32 (45.7%)	
Baseline stage group: Stage III	6 (17.1%)	19 (27.1%)	
ART duration, years	3.0 [1.5, 6.6]	3.2 [2.0, 9.0]	0.356
Exploratory comorbidity score	1.5 ± 1.3	1.5 ± 1.5	0.590
Exploratory comedication score	2.6 ± 1.4	2.1 ± 1.2	0.037
Chemotherapy	18 (51.4%)	31 (44.3%)	0.489
Antiangiogenic therapy	1 (2.9%)	8 (11.4%)	0.266
Previous tooth extraction at MRONJ lesion site	29 (82.9%)	58 (82.9%)	1.000

In the primary multivariable binary final-status model (*n* = 102; 68 healed; 34 not healed), surgical treatment was associated with higher odds of documented clinical soft tissue healing at the last recorded follow-up than non-surgical management (adjusted odds ratio [OR]: 11.12, 95% confidence interval [CI]: 4.12 to 33.35; *p* < 0.001), while cancer indication was associated with lower odds of documented healing than osteoporosis (adjusted OR: 0.23, 95% CI: 0.07 to 0.68; *p* = 0.011) (Table 5; Figure 4). The stage group was not independently associated with documented healing in this parsimonious model. Supplementary Figure S5 shows model-based predicted probabilities.

**Table 5:** Multivariable logistic regression model for documented clinical soft tissue healing at the last recorded follow-up.

Model	Predictor	Adjusted OR	95% CI	*P*
Primary model (standard logistic)	Surgical vs. non-surgical	11.12	4.12 to 33.35	< 0.001
Primary model (standard logistic)	Cancer vs. osteoporosis	0.23	0.07 to 0.68	0.011
Primary model (standard logistic)	Stage II vs. Stage 0/I	0.65	0.19 to 2.14	0.489
Primary model (standard logistic)	Stage III vs. Stage 0/I	1.31	0.28 to 6.31	0.731

CI: confidence interval; OR: odds ratio.

Model *n* = 102; healed = 68; not healed = 34. Unknown final outcomes were excluded. Reference categories: non-surgical management, osteoporosis indication, and Stage 0/I. Firth logistic regression was used as a small-sample sensitivity analysis.

**Figure 4:**
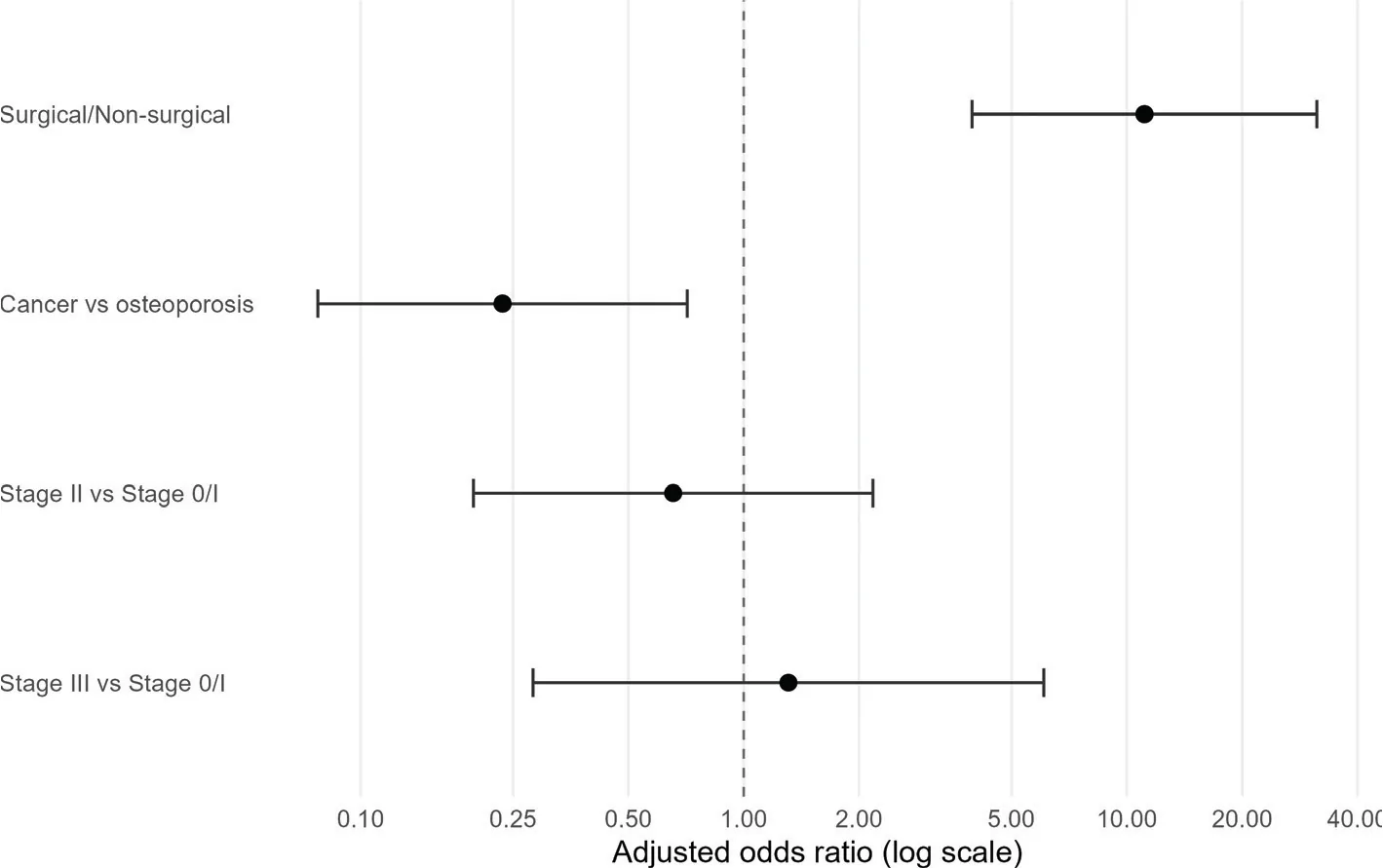
Adjusted associations with documented clinical soft tissue healing at the last recorded follow-up. Odds ratios and 95% confidence intervals are from the primary multivariable logistic regression model. Estimates describe associations with final documented clinical soft tissue status and not time to first healing.

Sensitivity analyses were directionally similar (Supplementary Tables S11 and S11B). After additional adjustment for sex, the treatment association remained positive (adjusted OR: 9.91, 95% CI: 3.56 to 30.57; *p* < 0.001). After adjustment for exploratory comedication burden, the corresponding estimate was 11.63 (95% CI: 4.23 to 35.81; *p* < 0.001), and after adjustment for both sex and exploratory comedication burden, it was 10.56 (95% CI: 3.73 to 33.28; *p* < 0.001). Excluding Stage 0 cases and Firth penalization also yielded directionally consistent estimates. When unknown outcomes were classified as no documented healing, the treatment estimate remained positive, whereas the cancer estimate attenuated and crossed the null. A secondary model accounting for duration of documented clinical observation yielded similar estimates for treatment and indication, while follow-up duration itself was not independently associated with documented healing.

In an additional exploratory analysis, the difference in comorbidity burden persisted after age adjustment, with cancer cases showing a lower count than osteoporosis cases (incidence rate ratio [IRR]: 0.59, 95% CI: 0.40–0.85; *p* = 0.005) (Table 6).

**Table 6:** Age-adjusted models for exploratory comorbidity burden by indication group.

Model	Term	IRR (95% CI)	*P*
Poisson model	Intercept	0.61 (0.14 to 2.64)	0.508
Poisson model	Cancer vs. osteoporosis	0.59 (0.40 to 0.85)	0.005
Poisson model	Age per 10 years	1.17 (0.97 to 1.40)	0.105
Poisson model + sex	Intercept	0.67 (0.16 to 2.88)	0.591
Poisson model + sex	Cancer vs. osteoporosis	0.53 (0.35 to 0.80)	0.003
Poisson model + sex	Age per 10 years	1.18 (0.99 to 1.41)	0.063
Poisson model + sex	Female sex	0.79 (0.53 to 1.16)	0.223

IRR: incidence rate ratio; CI: confidence interval.

## Discussion

This retrospective single-center study describes systemic profiles, oral findings, treatment patterns, and documented clinical soft tissue outcomes in a Norwegian cohort of 105 patients with MRONJ managed in a specialized university setting. Cancer- and osteoporosis-related MRONJ occurred in distinct systemic and pharmacological contexts, while showing broadly similar local oral manifestations, consistent with contemporary MRONJ position papers and the oncology bone-modifying-agent literature [1, 7, 14]. This study contributes a structured characterization of indication-specific case profiles and multivariable analysis, not an estimate of disease incidence or causal treatment effects.

Within this case-only cohort, shorter recorded antiresorptive treatment duration and diagnosis-driven oncological therapies distinguished cancer-related from osteoporosis-related MRONJ. At the same time, final documented clinical status and mortality differed by indication. These findings cannot be interpreted as incidence or relative risk estimates for treated populations because the cohort included only patients who had already developed MRONJ. Rather, they describe differences between two clinical profiles of MRONJ. Local dental disease and previous tooth extraction at the MRONJ lesion site were frequently recorded in both groups and should be interpreted as antecedent dental context rather than proof of a single causal trigger [30–32].

Tooth extraction and other dentoalveolar procedures were among the most frequently recorded antecedent dental events in this cohort, consistent with numerous retrospective and prospective reports [30–35]. Contemporary evidence also indicates that extraction undertaken within a structured preventive protocol, including control of local infection, atraumatic technique, alveoplasty where indicated, antimicrobial measures, and tension-free primary closure, may be associated with a low risk of subsequent MRONJ [36–39]. Many cases recorded possible clinical reasons or local dental conditions associated with extraction, although several conditions could coexist and completeness varied. Experimental studies also report that pre-existing dental infections lead to impaired post-extraction healing during antiresorptive exposure [40]. Reverse causality cannot be excluded; extraction may therefore indicate treatment of diseased teeth or early clinically unrecognized MRONJ rather than an independent primary cause [2, 16, 30–35]. Non-extraction presentations, including denture trauma and minor mucosal injury, are also compatible with a multifactorial local disease context [41, 42]. Studies of infection and bacterial diversity further support the relevance of the local disease environment [43, 44].

Systemically, the two indication groups differed in clinically predictable ways. Previous studies have examined systemic and treatment-related factors in oncology and osteoporosis populations, although their designs and populations differ [45–52]. Among cases with known final status, 68/102 (66.7%) had documented clinical soft tissue healing at the last recorded follow-up, within the range reported in heterogeneous clinical series [21, 53–62]. Patient-reported quality of life has also been evaluated separately from clinical healing [26, 63]. Documented healing was more frequent among patients receiving surgical treatment than among those managed non-surgically, and the adjusted association was directionally consistent across sensitivity analyses. However, these final-status analyses do not establish treatment efficacy. Treatment was not randomly allocated and likely reflected disease extent, systemic health, prognosis, clinician judgement, and suitability for surgery; therefore, it is important to interpret the findings as observational and hypothesis-generating [19, 22, 64–66]. At the same time, clinical worsening at the last recorded follow-up was uncommon among non-surgically managed patients, occurring in only 3 of 34 (8.8%) with known final outcome, and no worsening was recorded among 68 surgically treated patients with known final outcome. All patients in the present cohort had discontinued ART after their MRONJ diagnosis. The independent effect of this post-diagnosis discontinuation cannot be assessed because all patients followed this management, and exact timing and duration were unavailable. Ristow et al. also reported pausing antiresorptive therapy after MRONJ diagnosis [24]; differences in clinical course therefore cannot be attributed to withdrawal alone. The low proportion of documented worsening contrasts with reports of progression during conservative MRONJ management, including the study by Ristow et al. [24], and may reflect differences in case selection, local treatment protocols, follow-up duration, and outcome definitions (Supplementary Table S1B).

The exposure-duration patterns also require cautious interpretation. Cancer-related cases clustered around shorter but more intensive regimens, whereas osteoporosis-related cases had longer recorded treatment durations. These patterns are biologically plausible given the different schedules used in oncology and osteoporosis [48, 67, 68]. Nevertheless, this case-only cohort cannot estimate MRONJ risk in the underlying treated populations or separate the effects of indication, dose intensity, cumulative dose, adherence, and switching history [49–52]. The absence of validated performance-status measures further limits the interpretation of group differences in documented healing, as systemic fitness, survival, and opportunities for definitive treatment and follow-up may be interrelated [29, 62].

Stage 0 or non-exposed disease was uncommon (3/105, 2.9%). This likely reflects the referral of an already established MRONJ case to the Oral Surgery and Oral Medicine Department, as well as the diagnostic difficulty of non-specific Stage 0 presentations. Without exposed bone, early symptoms may be attributed to odontogenic or neuropathic conditions, and subtle radiographic changes may be overlooked or identified only after progression [69, 70]. Therefore, it is important to interpret stage-dependent conclusions cautiously. Comorbidity and comedication scores should likewise be understood as exploratory descriptive summaries rather than validated severity indices.

Mortality remains clinically relevant when interpreting final documented healing status. The lower proportion of documented healing in cancer-related cases may partly reflect patient death before mucosal closure or fewer opportunities for prolonged follow-up, rather than intrinsically poorer local repair. This may explain the pattern in our cohort; other studies have examined the adverse prognostic context of cancer-associated MRONJ and variation in its clinical course [29, 53, 71]. Treatment goals may therefore differ across indications. In oncology, early coordination between oncology and oral health services remains important for patients receiving high-intensity antiresorptive therapy with chemotherapy or antiangiogenic agents [14, 18, 72, 73]. In osteoporosis, systematic dental and periodontal assessment, patient education, and careful planning of invasive dental treatment remain important during long-term therapy [28].

This study has several limitations. Its retrospective, single-center, case-only design precludes causal inference and does not permit estimation of MRONJ incidence or risk among the treated populations. Possible clinical reasons or local dental conditions associated with extraction were recorded, but exact extraction dates, dentist-level decision documentation, and biopsy confirmation at extraction were unavailable; clinically unrecognized MRONJ at extraction cannot therefore be excluded. Treatment comparisons are vulnerable to confounding by indication. The dataset lacked validated performance-status measures and sufficiently structured patient-level details on antibiotics, antimicrobial mouth rinses, and the timing and duration of ART discontinuation after MRONJ diagnosis. All patients discontinued ART after diagnosis, so its independent effect could not be assessed. Serial radiographic findings were not available in a standardized analytical dataset; clinical mucosal coverage may therefore coexist with persistent radiographic abnormalities or residual non-exposed disease [6, 42, 70]. Stage 0 was uncommon and may have been under-ascertained.” Three Stage 0 cases were recorded; the limitation is possible under-ascertainment, not complete absence of captured cases. Finally, the exact date of the first healing was unavailable, so the study evaluated the final documented clinical soft tissue status rather than a conventional time-to-first-healing endpoint. Local findings and invasive procedures should therefore be interpreted as recorded clinical context, not as independently established causal factors [2, 16, 30–35].

The high proportion of cases with a recorded history of previous tooth extraction at the MRONJ lesion site, particularly in the osteoporosis group, indicates an opportunity to strengthen preventive oral-care pathways through coordinated dental assessment, management of local infections and diseases, and structured protocols for necessary invasive procedures before and during antiresorptive therapy [31, 36–39, 74].

## Conclusions

Among patients with MRONJ, cancer-related and osteoporosis-related cases showed broadly similar local oral disease patterns but distinct systemic and treatment-exposure profiles. A recorded history of previous tooth extraction at the MRONJ lesion site was common, particularly in osteoporosis-related MRONJ, but the retrospective data did not permit verification of the extraction indication or precise temporal attribution. Documented clinical soft-tissue healing at the last recorded follow-up was less frequent in cancer-related cases, while surgical treatment was associated with a higher likelihood of documented healing. These findings should be interpreted cautiously because treatment allocation was non-random, exact first-healing dates were unavailable, and radiographic resolution was not assessed. Clinical worsening was uncommon among non-surgically treated patients and was not observed after surgical treatment; however, this observation remains descriptive and should not be interpreted as evidence of treatment superiority.

## Supplementary Material



## Data Availability

The data may be made available by the corresponding author upon reasonable request, subject to applicable Norwegian privacy legislation, institutional approval, and approval by the Regional Committee for Medical and Health Research Ethics.

## Supplementary information

Supplementary Methods, Supplementary Results, Supplementary Tables S1-S12 (including S1B and S11B), and Supplementary Figures S1–S7 accompany this paper.
