# Supplementary material for: Medication-related osteonecrosis of the jaw in cancer and osteoporosis patients: a retrospective seven-year single-center Norwegian cohort study of clinical profiles and documented soft tissue healing
Source: Acta Odontol Scand. 2026 Oct 9;85:46983. doi: 10.2340/aos.v85.46983 (PMC13660455; doi:10.2340/aos.v85.46983)
Supplement: Supplementary file 1 [file AOS-85-46983-s1.pdf]

Supplementary material has been published as submitted. It has not been copyedited or typeset by Acta Odontologica Scandinavica.

Supplementary Material:

Supplementary Materials and Methods

S1. Variable definitions and coding

Variables were extracted from the original hospital-based dataset and recorded for the revised analyses. Unless otherwise stated, binary variables were coded as 0 = absent and 1 = present at the time of MRONJ diagnosis. All 105 patients with verified MRONJ were included, and all consented to inclusion in the study and registration of their clinical data for research.

S1.1 Systemic comorbidities

Recorded systemic comorbidities comprised diabetes mellitus, cardiovascular disease, hypertension, hypercholesterolaemia, hypothyroidism, rheumatoid arthritis, polymyalgia rheumatica, chronic obstructive pulmonary disease, asthma, and a residual category of other clinically relevant systemic disorders. Smoking status was coded separately as current smoker versus non-current smoker and was used descriptively.

S1.2 Local oral conditions

Local oral variables included generalised marginal bone loss/periodontitis, apical periodontitis at the lesion site, tooth mobility, previous endodontic treatment at the lesion site, deep caries near the lesion, denture trauma, widened periodontal ligament space, sclerotic lamina dura, oral tori, and generalised dental attrition/bruxism. These variables were used mainly for descriptive analyses and secondarily for exploratory analyses of local disease context.

S1.3 Concomitant medications

Concomitant systemic medications recorded at or around MRONJ diagnosis included corticosteroids, antihypertensives, lipid-lowering agents, chemotherapy, proton pump inhibitors, antidepressants, asthma medication, antiangiogenic drugs, radium-223, and other long-term medications such as antithrombotics, antidiabetics, antiepileptics, antiparkinsonian drugs, psycholeptics, gastrointestinal drugs, vitamin D, vitamin B derivatives, and calcium supplements. The total number of concomitant medications was also calculated for descriptive use.

S1.4 Construction of burden scores

Two simple exploratory burden scores were constructed to summarise systemic background. The comorbidity score (theoretical range 0–9) was the sum of diabetes, cardiovascular disease, hypertension, hypercholesterolaemia, COPD, asthma, rheumatoid arthritis, hypothyroidism, and other comorbidities. The comedication score (theoretical range 0–9) was the sum of corticosteroids, chemotherapy, antiangiogenic drugs, antihypertensives, proton pump inhibitors, lipid-lowering agents, antidepressants, antihormonal therapy, and asthma medication. Other chronic medications were described separately and did not contribute to this score. The stored comedication score matched the original R calculation. Missing or free-text blanks in component variables were treated as zero by the original row-sum procedure; the score is therefore interpreted as an exploratory burden measure. Group-specific systemic comorbidities and detailed concomitant medication distributions are reported in Supplementary Tables S2 and S3. For descriptive analyses, both scores were also categorised as 0–1, 2–3, and ≥4. In sensitivity analyses of indication models, chemotherapy and antiangiogenic therapy were excluded from the predictor set to reduce the influence of diagnosis-driven oncological treatment variables. Multiple correspondence analysis (MCA) followed by k-means clustering was used to visualize the recorded patient profiles descriptively. Indication group was included among the input

variables, so the analysis did not independently validate indication-specific clusters (Supplementary Figure S1).

S1.5 Exposure, treatment, and endpoint constraints

The primary group was cancer-related versus osteoporosis-related MRONJ. A proxy high-dose versus low-dose BMA classification was derivable from indication, agent, route, and dosing frequency, but it was concordant with indication in this cohort and therefore did not provide an independent sensitivity analysis. The source treatment variable retained four categories: non-surgical management, surgical treatment, sequestrectomy, and other surgery. Surgical treatment was conservative and included sequestrectomy or marginal bone resection followed by primary soft tissue closure using local flaps; no segmental resections were performed in this patient cohort. For the primary healing analysis, the three operative categories were combined as surgical treatment; detailed patient-level resection extent, perioperative antibiotics, antimicrobial mouth rinses, local wound care, postoperative regimens, and prophylactic drug-holiday timing were not available in a standardised form. The clinical endpoint was final documented soft-tissue healing status, not time to first healing, and no predefined minimum follow-up threshold was applied. Radiographic healing was not assessable.

S2. Model diagnostics and technical details

S2.1 Logistic regression model for indication

The main multivariable logistic regression model used indication for antiresorptive therapy (cancer = 1, osteoporosis = 0) as the dependent variable. Covariates were age, sex, duration of antiresorptive therapy (ART), COPD, other systemic comorbidity, chemotherapy, systemic corticosteroids, proton pump inhibitors, and recorded tooth extraction at the MRONJ site. Model discrimination was assessed by the area under the receiver operating characteristic curve (AUC), and calibration was examined by comparing observed and predicted probabilities across deciles of fitted risk. These figures describe apparent in-sample performance; no internal or external validation was performed [Supplementary Figures S3 and S4]. Reduced and penalised sensitivity models are presented in Supplementary Tables S4 and S5.

S2.2 Logistic models for documented clinical soft tissue healing at the last recorded follow-up

Documented clinical soft tissue healing at the last recorded follow-up was analysed as a binary endpoint. The primary model included treatment group, indication group, and baseline stage group. Standard logistic regression was fitted first, with Firth-penalised logistic regression treated as a small-sample sensitivity analysis. Additional sensitivity analyses examined adjustment for sex, exploratory comedication burden, both variables together, unknown-outcome handling, exclusion of Stage 0, age, ART duration, and follow-up duration as a duration-of-observation variable. The high-dose versus low-dose proxy was concordant with indication and was therefore not treated as an independent sensitivity analysis. Unknown final outcomes were excluded from the primary model and classified as no documented healing in sensitivity analysis. Because the model was deliberately parsimonious, residual confounding by performance status and unmeasured clinical selection remains possible. Summary diagnostics and analytic sample flow are shown in Supplementary Tables S9 and S10.

S2.3 Missing data

Missingness was low for most variables used in the revised models. The primary healing model excluded three cases with unknown final clinical status; the secondary follow-up-adjusted analysis additionally required available follow-up duration. No imputation was performed [Supplementary Table S6].

Supplementary Results

S3. Advanced and sensitivity models

S3.1 Firth-penalised logistic regression for indication

Because several predictors showed sparse data patterns and quasi-complete separation between cancer-related and osteoporosis-related MRONJ, a Firth-penalised logistic regression model was fitted as a sensitivity analysis. The retained model included age, sex, antiresorptive therapy duration, COPD, other systemic comorbidity, chemotherapy, antiangiogenic therapy, systemic corticosteroids, proton pump inhibitors, and recorded tooth extraction at the MRONJ site.

The penalised analysis supported the direction of the main indication findings. Shorter antiresorptive therapy duration remained strongly associated with cancer indication, and male sex was associated with higher odds of cancer indication. Chemotherapy also remained strongly associated with cancer indication. Antiangiogenic therapy showed a large point estimate with a very wide confidence interval, consistent with sparse exposure and near-separation in the dataset. Full model results are presented in [Supplementary Table S5].

S3.2 Secondary ascertainment analyses for final clinical status

Because follow-up duration reflected time from treatment start to the last recorded visit rather than a baseline covariate, it was examined only in secondary ascertainment models. A model including follow-up per 6 months produced directionally similar treatment and indication estimates, while follow-up duration itself was not independently associated with documented healing [Supplementary Table S11].

Model-based predicted probabilities from the primary logistic model by treatment and indication are shown in Supplementary Figure S5. Observed documented-healing proportions by treatment group are shown in Supplementary Figure S6.

S3.3 Multiple correspondence analysis and clustering

MCA was performed using categorical representations of key clinical variables, including indication group, route of administration, MRONJ stage, selected comorbidities, concomitant medications, and treatment characteristics. The first two MCA dimensions were used as input to a k-means clustering procedure with $k = 2$. Because indication group was included among the input variables, the analysis was interpreted only as a descriptive visualisation and not as independent confirmation of indication-specific clusters.

One cluster was enriched for cancer patients receiving high-potency intravenous ART, chemotherapy, antiangiogenic therapy, and higher comedication burden. The other contained a higher proportion of osteoporosis patients with longer ART duration, higher exploratory comorbidity burden, and more extraction-related lesions. The clusters overlapped and were not intended for clinical classification [Supplementary Figure S1].

S4. Missing data

Missingness remained low in the revised analysis dataset. The main analysis-scale missing variables were follow-up time (2 patients), ART duration in months (1 patient), pain score (2 patients), and death status or time variables (4–6 patients, depending on the field). No imputation was performed because complete-case modelling remained feasible and the proportion of missingness was small [Supplementary Table S6]. Current smoking status was missing in 16 patients (15.2%) (Supplementary Table S6).

S5. Stage distribution and Stage 0 under-capture

Stage II was the most common presentation in both indication groups. Stage 0 or non-exposed disease was rare (3 cases overall), which is consistent with likely under-capture of non-exposed

disease in retrospective tertiary-care records and limits how confidently stage-specific findings can be generalised to early MRONJ presentations [Supplementary Table S7].

S6. Extraction-related variables and reverse-causality constraints

Extraction at the lesion site was recorded in 87 patients. Among these cases, several non-mutually-exclusive variables captured possible clinical reasons or local dental conditions associated with extraction, including mobile teeth, previous endodontic treatment/failure, fracture or root-remnant findings, deep caries, apical periodontitis, and generalised marginal bone loss/periodontitis. Completeness varied, and these variables do not constitute verified extraction indications. Exact extraction dates, dentist-level decision documentation, biopsy confirmation at extraction, and structured data on preventive precautions were unavailable. Reverse causality and clinically unrecognised MRONJ at extraction therefore cannot be excluded [Supplementary Table S8; Supplementary Figure S7].

S7. Binary healing model diagnostics

The primary binary final-status model included 102 cases with known final status, of whom 68 had documented healing and 34 did not. No complete or quasi-complete separation was detected; the maximum standard error was modest (0.783), and discrimination was acceptable (AUC 0.816; Brier score 0.146). Summary diagnostics and analytic sample flow are shown in Supplementary Tables S9 and S10. Observed outcome proportions by treatment group are shown in Supplementary Figure S6.

S8. Sensitivity analyses

Sensitivity analyses were directionally consistent with the primary binary model. The treatment association remained positive after additional adjustment for sex, exploratory comedication burden, and both variables together. The association also remained directionally consistent after Stage 0 exclusion, Firth penalisation, unknown-outcome reclassification, and adjustment for follow-up duration; the cancer estimate attenuated in models including sex and in the unknown-outcome reclassification analysis [Supplementary Tables S11 and S11B]. Influence and leave-one-out analyses did not identify any single observation that changed the substantive interpretation of the treatment estimate.

S9. Treatment-selection model

Measured baseline differences between non-surgical and surgical treatment were present. The exploratory treatment-selection model did not identify a dominant measured predictor, but residual confounding by clinical judgement, performance status, prognosis, surgical suitability, and other unmeasured factors remains possible [Supplementary Table S12].

S10. Charlson feasibility assessment

A formal Charlson Comorbidity Index could not be validly reconstructed from the available dataset because several required diagnostic categories were absent, aggregated, or insufficiently specific.

S11. Availability of additional retrospective data elements

A focused audit showed partial availability of possible clinical reasons or local dental conditions associated with extraction, but exact extraction dates and biopsy confirmation were unavailable. ART discontinuation in all patients and the clinical radiography protocol were available as cohort-level author clarifications, whereas exact cessation timing, patient-level regimens, serial radiographic findings, and exact first-healing dates were not available in structured form.

Supplementary Table S1. Expanded clinical characteristics, MRONJ stage and localisation, treatment group, follow-up, and outcomes by indication group.

Characteristic	Cancer	Osteoporosis	P value
MRONJ stage: Stage 0	1 (1.8%)	2 (4.1%)	0.380
MRONJ stage: Stage I	12 (21.4%)	13 (26.5%)	
MRONJ stage: Stage II	26 (46.4%)	26 (53.1%)	
MRONJ stage: Stage III	17 (30.4%)	8 (16.3%)	
Lesion site: Maxilla	23 (41.1%)	14 (28.6%)	0.208
Lesion site: Mandible	29 (51.8%)	27 (55.1%)	
Lesion site: Both jaws	4 (7.1%)	8 (16.3%)	
Treatment group: Non-surgical	21 (37.5%)	14 (28.6%)	0.333
Treatment group: Surgical	35 (62.5%)	35 (71.4%)	
Follow-up from treatment start, months	11.0 [6.8, 17.5]	8.0 [5.0, 15.5]	0.064
Final status available for healing analysis	56 (100.0%)	46 (93.9%)	0.098
Documented soft tissue healing among patients with known final status	31/56 (55.4%)	37/46 (80.4%)	0.008
Not healed among patients with known final status	25/56 (44.6%)	9/46 (19.6%)	
Death recorded during follow-up	40/55 (72.7%)	15/46 (32.6%)	<0.001

Supplementary Table S1B. Final clinical outcome by treatment group. The full outcome distribution uses all 35 non-surgical and 70 surgical patients; healing and worsening comparisons use only patients with a known final outcome.

Clinical outcome or comparison	Non-surgical (n = 35)	Surgical (n = 70)	P value
Soft tissue healed	11 (31.4%)	57 (81.4%)	
Improved	5 (14.3%)	6 (8.6%)	
No change	15 (42.9%)	5 (7.1%)	
Worsened	3 (8.6%)	0 (0.0%)	
Unknown / not recorded	1 (2.9%)	2 (2.9%)	
Overall final-outcome distribution (all patients)			<0.001
Known final clinical outcome	34/35 (97.1%)	68/70 (97.1%)	1.000
Documented soft tissue healing among patients with known final status	11/34 (32.4%)	57/68 (83.8%)	<0.001
Not healed among patients with known final status	23/34 (67.6%)	11/68 (16.2%)	
Worsened among patients with known final status	3/34 (8.8%)	0/68 (0.0%)	0.035

Categorical data are presented as n (%) and follow-up as median [IQR]. For MRONJ stage, lesion site, and treatment group, the overall p-value is shown once for the complete distribution. Healing percentages use cases with known final status as the denominator. Stage 0 denotes non-exposed MRONJ according to AAOMS staging.

Supplementary Table S2. Selected systemic comorbidities and comedications by indication group in cancer-related and osteoporosis-related MRONJ.

Characteristic	Cancer	Osteoporosis	P value
Diabetes mellitus	6 (10.7%)	5 (10.2%)	0.932
Cardiovascular disease	4 (7.1%)	8 (16.7%)	0.130
Hypercholesterolaemia	13 (23.2%)	19 (38.8%)	0.084
COPD	2 (3.6%)	9 (18.4%)	0.014
Asthma	0 (0.0%)	7 (14.3%)	0.004
Rheumatoid arthritis	0 (0.0%)	10 (20.4%)	<0.001
Hypothyroidism	7 (12.5%)	4 (8.2%)	0.469
Systemic corticosteroids	28 (50.0%)	18 (36.7%)	0.172
Antiangiogenic drugs	9 (16.1%)	0 (0.0%)	0.003
Chemotherapy	46 (82.1%)	3 (6.1%)	<0.001
Radium-223	4 (7.1%)	0 (0.0%)	0.121
Lipid-lowering drugs	14 (25.0%)	18 (36.7%)	0.192
Proton pump inhibitors	13 (23.2%)	11 (22.4%)	0.926
Antihypertensive drugs	21 (37.5%)	23 (47.9%)	0.284
Antidepressants	7 (12.5%)	13 (26.5%)	0.068

P-values are from Pearson chi-square or Fisher exact tests selected according to the expected-cell rule specified in the Methods.

Supplementary Table S3. Detailed concomitant systemic medications by indication group in cancer-related and osteoporosis-related MRONJ.

Comedication	Cancer	Osteoporosis	P value
Corticosteroids	28 (50%)	18 (37%)	0.172
Antihypertensive	21 (38%)	23 (47%)	0.284
Lipid-lowering	14 (25%)	18 (37%)	0.192
Chemotherapy	46 (82%)	3 (6%)	<0.001
Aromatase inhibitors	12 (21%)	0 (0%)	<0.001
Immunosuppressants	8 (14%)	0 (0%)	0.007
Protein kinase inhibitors	5 (9%)	0 (0%)	0.059
Monoclonal antibodies	3 (5%)	0 (0%)	0.246
Pyrimidine analogs	3 (5%)	0 (0%)	0.246
Plant alkaloids	6 (11%)	0 (0%)	0.029
Proton pump inhibitors (PPI)	13 (23%)	11 (22%)	0.926
Antidepressants	7 (13%)	13 (27%)	0.068
Antiasthmatics	5 (9%)	12 (24%)	0.014
Antiangiogenic	9 (16%)	0 (0%)	0.003
Radium 223	4 (7%)	0 (0%)	0.121
Analgesia	15 (27%)	20 (41%)	0.150
Gastrointestinal	8 (14%)	3 (6%)	0.213
Antithrombotic	15 (27%)	22 (45%)	0.066
Antidiabetic	2 (4%)	3 (6%)	0.662
Antiepileptic	4 (7%)	7 (14%)	0.340
Antiparkinsonian	1 (2%)	1 (2%)	1.000
Psycholeptics	8 (14%)	14 (29%)	0.094
Vitamin D	2 (4%)	3 (6%)	0.662
Vitamin B	3 (5%)	5 (10%)	0.469
Calcium	15 (27%)	20 (41%)	0.150

Total number of drugs used

Comedication	Cancer	Osteoporosis	P value
0	0 (0%)	2 (4%)	0.215
< 5	25 (45%)	24 (49%)	0.698
5 - 10	27 (48%)	20 (41%)	0.555
> 10	4 (7%)	3 (6%)	1.000

P-values are from Pearson chi-square or Fisher exact tests selected according to the expected-cell rule specified in the Methods where structured patient-level variables were available; detailed medication subclass rows not represented in the structured source should be interpreted descriptively.

Supplementary Table S4. Sensitivity analysis of the indication model after exclusion of diagnosis-driven treatment variables.

Variable	Odds ratio	95% CI	P value
Age (per 10 years)	0.570	0.26 to 1.13	0.120
Female sex (vs male)	0.100	0.01 to 0.54	0.013
ART duration (per year)	0.640	0.48 to 0.78	<0.001
COPD	0.030	0.00 to 0.21	0.003
Other systemic comorbidity	0.540	0.07 to 3.54	0.521
Systemic corticosteroids	3.260	0.71 to 18.52	0.147
Proton pump inhibitor	0.970	0.22 to 4.29	0.966
Recorded extraction at the lesion site	0.100	0.01 to 0.79	0.053

Note: Confidence intervals are profile-likelihood intervals, whereas p-values are Wald tests; values close to the 0.05 threshold may therefore not correspond exactly.

Supplementary Table S5. Firth-penalised logistic regression sensitivity analysis for cancer versus osteoporosis as the indication for antiresorptive therapy.

Variable	Odds ratio	95% CI	P value
Age (per 10 years)	1.290	0.52 to 3.50	0.591
Female sex (vs male)	0.080	0.00 to 0.61	0.012
ART duration (per year)	0.650	0.42 to 0.85	<0.001
COPD	0.180	0.01 to 1.82	0.161
Other systemic comorbidity	0.590	0.03 to 6.32	0.689
Chemotherapy	21.750	4.40 to 225.39	<0.001
Antiangiogenic therapy	39.230	0.35 to 71656.83	0.193
Systemic corticosteroids	0.810	0.12 to 4.60	0.817
Proton pump inhibitor	1.320	0.24 to 8.00	0.749
Recorded extraction at the lesion site	0.380	0.03 to 2.74	0.346

Supplementary Table S6. Variable-level missingness in the revised analysis dataset.

Variable or field	Missing/unknown n	Missing/unknown %
Final clinical status coded as unknown/not recorded	3	2.9
Follow-up duration from treatment start	2	1.9
ART duration	1	1.0
Current smoking status	16	15.2
Death status	4	3.8
Time from MRONJ diagnosis to death	6	5.7
Pain score	2	1.9
Deep caries at lesion site	3	2.9
Apical periodontitis at lesion site	1	1.0
Endodontic treatment/failure at lesion site	1	1.0
Denture trauma	1	1.0
Cardiovascular disease	1	1.0
Antihypertensive medication	1	1.0

The source pain-score coding included an out-of-range value that could not be resolved retrospectively; pain was therefore not retained in the final comparative table. No imputation was performed.

Supplementary Table S7. MRONJ stage distribution by indication group.

Indication	Stage	n	Within-group percentage (%)
Osteoporosis	Stage 0	2	4.1
Osteoporosis	Stage I	13	26.5
Osteoporosis	Stage II	26	53.1
Osteoporosis	Stage III	8	16.3
Cancer	Stage 0	1	1.8
Cancer	Stage I	12	21.4
Cancer	Stage II	26	46.4
Cancer	Stage III	17	30.4

Supplementary Table S8. Recorded possible reasons or local dental conditions associated with extraction among patients with an extraction recorded at the MRONJ lesion site**.**

Possible reason or local dental condition	Cancer	Osteoporosis	Cancer unknown n	Osteoporosis unknown n
Mobile tooth	11/40 (27.5%)	12/47 (25.5%)	0	0
Prior endodontic treatment/failure	11/40 (27.5%)	9/47 (19.1%)	0	1
Tooth/crown fracture or root remnants	3/40 (7.5%)	8/47 (17.0%)	0	0
Deep caries	6/40 (15.0%)	4/47 (8.5%)	0	1
Apical periodontitis	14/40 (35.0%)	9/47 (19.1%)	0	1
Generalised marginal bone loss/periodontitis	24/40 (60.0%)	31/47 (66.0%)	0	0
No selected condition recorded	6/40 (15.0%)	12/47 (25.5%)		

Supplementary Table S9. Summary diagnostics for the primary binary final-status healing model.

metric	value
Primary model sample size	102.0
Primary model healed	68.0

Primary model not healed	34.0
Primary model coefficient count excluding intercept	4.0
Smaller outcome category per coefficient	8.5
Any infinite coefficient	0.0
Maximum standard error	0.7831
Complete/quasi-complete separation detected	0.0
Potentially influential observation identified	1
Maximum Cook distance	0.1267
Maximum leverage	0.0987
Brier score	0.146
AUC	0.8157

The primary model included 68 healed and 34 non-healed cases with four coefficients excluding the intercept. One potentially influential observation was identified, but influence and leave-one-out analyses did not materially alter the treatment estimate or its substantive interpretation.

Supplementary Table S10. Logistic-model diagnostics and analytic sample flow.

Section	Item	Value
Study flow	Verified MRONJ cases in source cohort	105
Study flow	Diagnosed MRONJ cases excluded	0
Study flow	Cancer-related cases	56
Study flow	Osteoporosis-related cases	49
Primary endpoint	Known final clinical status	102
Primary endpoint	Unknown/not recorded final status	3
Primary analysis	Complete-case sample	102
Primary analysis	Healed	68
Primary analysis	Not healed	34
Secondary analysis	Available follow-up duration	103
Study flow	All included patients consented to inclusion and registration of their clinical data for research	105

Supplementary Table S11. Sensitivity analyses for documented clinical soft tissue healing.

Model	Predictor	Adjusted OR	95% CI	P value
Sensitivity 1: Unknown outcomes as no documented healing	Intercept	1.09	0.33 to 3.66	0.892
Sensitivity 1: Unknown outcomes as no documented healing	Surgical vs non-surgical	9.58	3.75 to 26.56	<0.001
Sensitivity 1: Unknown outcomes as no documented healing	Cancer vs osteoporosis	0.39	0.14 to 1.01	0.058
Sensitivity 1: Unknown outcomes as no documented healing	Stage II vs Stage 0/I	0.60	0.18 to 1.87	0.386
Sensitivity 1: Unknown outcomes as no documented healing	Stage III vs Stage 0/I	0.78	0.19 to 3.14	0.721
Sensitivity 2: Exclude Stage 0	Intercept	1.15	0.30 to 4.52	0.836
Sensitivity 2: Exclude Stage 0	Surgical vs non-surgical	11.13	4.08 to 33.66	<0.001
Sensitivity 2: Exclude Stage 0	Cancer vs osteoporosis	0.27	0.08 to 0.78	0.020
Sensitivity 2: Exclude Stage 0	Stage II vs Stage 0/I	0.71	0.19 to 2.42	0.585
Sensitivity 2: Exclude Stage 0	Stage III vs Stage 0/I	1.36	0.28 to 6.71	0.702
Sensitivity 3: Add age per 10 years	Intercept	6.46	0.12 to 500.65	0.373
Sensitivity 3: Add age per 10 years	Surgical vs non-surgical	11.21	4.13 to 33.87	<0.001
Sensitivity 3: Add age per 10 years	Cancer vs osteoporosis	0.20	0.06 to 0.63	0.008
Sensitivity 3: Add age per 10 years	Stage II vs Stage 0/I	0.70	0.20 to 2.38	0.574
Sensitivity 3: Add age per 10 years	Stage III vs Stage 0/I	1.43	0.30 to 6.96	0.650
Sensitivity 3: Add age per 10 years	Age per 10 years	0.81	0.47 to 1.36	0.426
Sensitivity 4: Add ART duration per year	Intercept	1.51	0.31 to 7.60	0.611
Sensitivity 4: Add ART duration per year	Surgical vs non-surgical	10.85	3.99 to 32.81	<0.001

Sensitivity 4: Add ART duration per year	Cancer vs osteoporosis	0.21	0.06 to 0.74	0.018
Sensitivity 4: Add ART duration per year	Stage II vs Stage 0/I	0.63	0.18 to 2.06	0.449
Sensitivity 4: Add ART duration per year	Stage III vs Stage 0/I	1.32	0.29 to 6.38	0.719
Sensitivity 4: Add ART duration per year	ART duration per year	0.99	0.88 to 1.14	0.864
Sensitivity 5: Firth logistic regression	Intercept	1.30	0.40 to 4.47	0.666
Sensitivity 5: Firth logistic regression	Surgical vs non-surgical	9.49	3.68 to 26.81	<0.001
Sensitivity 5: Firth logistic regression	Cancer vs osteoporosis	0.26	0.08 to 0.72	0.009
Sensitivity 5: Firth logistic regression	Stage II vs Stage 0/I	0.68	0.21 to 2.12	0.509
Sensitivity 5: Firth logistic regression	Stage III vs Stage 0/I	1.28	0.29 to 5.79	0.741
Sensitivity 6: Add follow-up per 6 months	Intercept	1.50	0.40 to 5.91	0.550
Sensitivity 6: Add follow-up per 6 months	Surgical vs non-surgical	11.67	4.24 to 35.99	<0.001
Sensitivity 6: Add follow-up per 6 months	Cancer vs osteoporosis	0.24	0.07 to 0.71	0.013
Sensitivity 6: Add follow-up per 6 months	Stage II vs Stage 0/I	0.66	0.19 to 2.15	0.491
Sensitivity 6: Add follow-up per 6 months	Stage III vs Stage 0/I	1.37	0.29 to 6.72	0.688
Sensitivity 6: Add follow-up per 6 months	Follow-up per 6 months	0.93	0.70 to 1.25	0.610
Sensitivity 6b: Add log-transformed follow-up	Intercept	0.85	0.11 to 6.20	0.873
Sensitivity 6b: Add log-transformed follow-up	Surgical vs non-surgical	10.89	4.02 to 32.71	<0.001
Sensitivity 6b: Add log-transformed follow-up	Cancer vs osteoporosis	0.23	0.07 to 0.66	0.009
Sensitivity 6b: Add log-transformed follow-up	Stage II vs Stage 0/I	0.65	0.19 to 2.14	0.486
Sensitivity 6b: Add log-transformed follow-up	Stage III vs Stage 0/I	1.22	0.26 to 6.00	0.800
Sensitivity 6b: Add log-transformed follow-up	log1p(follow-up months)	1.23	0.61 to 2.57	0.566

The derived high-dose/low-dose proxy was concordant with indication in this cohort and therefore did not constitute an independent sensitivity analysis.

Supplementary Table S11B. Confounding sensitivity models for documented clinical soft tissue healing. Conventional logistic regression models used cases with known final clinical outcome. Model 0 included treatment group, indication group, and stage group; Model 1 additionally included sex; Model 2 additionally included exploratory comedication burden; Model 3 included both sex and comedication burden; and Model 4 additionally included age and exploratory comorbidity burden. Estimates describe observational associations and should not be interpreted causally. Firth-penalised sensitivity estimates were directionally consistent.

Model	Predictor	Adjusted OR	95% CI	P value	n
Model 0	Surgical vs non-surgical	11.12	4.12 to 33.35	<0.001	102
Model 0	Cancer vs osteoporosis	0.23	0.07 to 0.68	0.011	102
Model 0	Stage II vs Stage 0/I	0.65	0.19 to 2.14	0.489	102
Model 0	Stage III vs Stage 0/I	1.31	0.28 to 6.31	0.731	102
Model 1	Surgical vs non-surgical	9.91	3.56 to 30.57	<0.001	102
Model 1	Cancer vs osteoporosis	0.39	0.11 to 1.33	0.135	102
Model 1	Stage II vs Stage 0/I	0.64	0.18 to 2.17	0.477	102
Model 1	Stage III vs Stage 0/I	1.16	0.24 to 5.64	0.854	102
Model 1	Female vs male	3.19	1.04 to 10.25	0.044	102
Model 2	Surgical vs non-surgical	11.63	4.23 to 35.81	<0.001	102
Model 2	Cancer vs osteoporosis	0.22	0.06 to 0.66	0.010	102
Model 2	Stage II vs Stage 0/I	0.64	0.18 to 2.10	0.469	102
Model 2	Stage III vs Stage 0/I	1.33	0.28 to 6.48	0.718	102
Model 2	Exploratory comedication score (per point)	1.11	0.73 to 1.74	0.624	102
Model 3	Surgical vs non-surgical	10.56	3.73 to 33.28	<0.001	102
Model 3	Cancer vs osteoporosis	0.37	0.10 to 1.32	0.129	102
Model 3	Stage II vs Stage 0/I	0.62	0.17 to 2.14	0.459	102
Model 3	Stage III vs Stage 0/I	1.18	0.24 to 5.82	0.837	102
Model 3	Female vs male	3.80	1.17 to 13.60	0.031	102
Model 3	Exploratory comedication score (per point)	1.26	0.81 to 2.01	0.317	102
Model 4	Surgical vs non-surgical	11.21	3.88 to 36.62	<0.001	102
Model 4	Cancer vs osteoporosis	0.28	0.06 to 1.12	0.080	102
Model 4	Stage II vs Stage 0/I	0.67	0.18 to 2.39	0.538	102
Model 4	Stage III vs Stage 0/I	1.27	0.26 to 6.39	0.770	102
Model 4	Age per 10 years	0.71	0.39 to 1.23	0.228	102
Model 4	Female vs male	4.65	1.34 to 18.41	0.020	102
Model 4	Exploratory comorbidity score (per point)	0.89	0.51 to 1.55	0.682	102

Model 4	Exploratory comedication score (per point)	1.41	0.81 to 2.53	0.230	102

Supplementary Table S12. Treatment-selection model for surgical versus non-surgical management.

Characteristic	Adjusted OR	95% CI	P value
Intercept	1.93	0.05 to 80.49	0.720
Age per 10 years	1.00	0.62 to 1.58	0.989
Female vs male	2.37	0.86 to 6.78	0.099
Cancer vs osteoporosis	0.97	0.23 to 4.38	0.970
Stage II vs Stage 0/I	0.83	0.29 to 2.30	0.728
Stage III vs Stage 0/I	1.53	0.42 to 5.85	0.517
Exploratory comorbidity score (per point)	1.10	0.74 to 1.68	0.645
Exploratory comedication score (per point)	0.73	0.46 to 1.15	0.183
Chemotherapy	1.31	0.34 to 5.18	0.689

The model was exploratory and included only the listed variables; absence of a statistically significant predictor does not exclude residual or unmeasured determinants of treatment allocation.

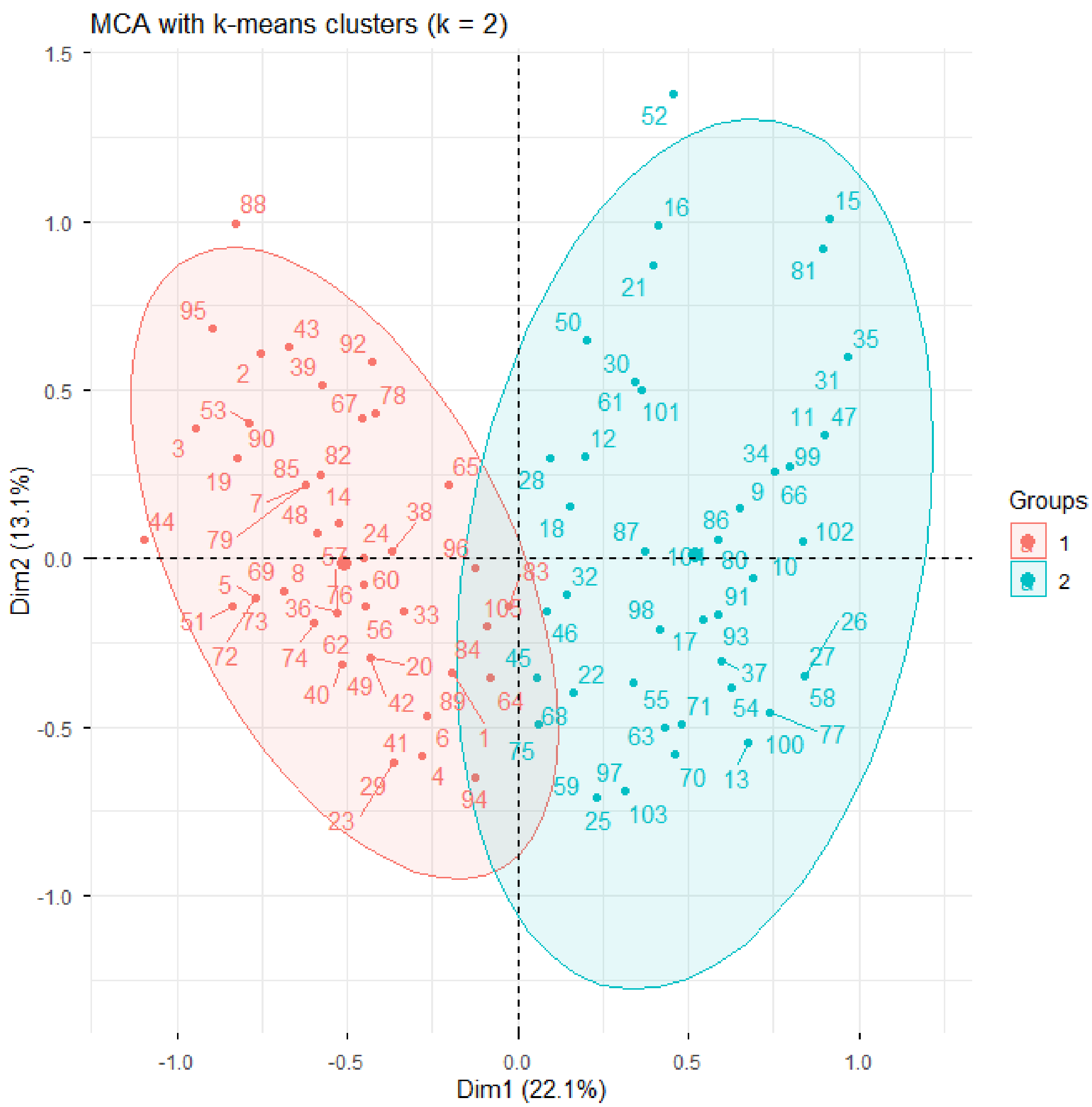

Supplementary Figure S1. Descriptive multiple correspondence analysis (MCA) of MRONJ case profiles. Each dot represents one patient; patients plotted close together have more similar overall profiles. Indication group was included among the input variables, so the partially overlapping clouds should be interpreted as a descriptive visualisation rather than independent confirmation of indication-specific clusters.

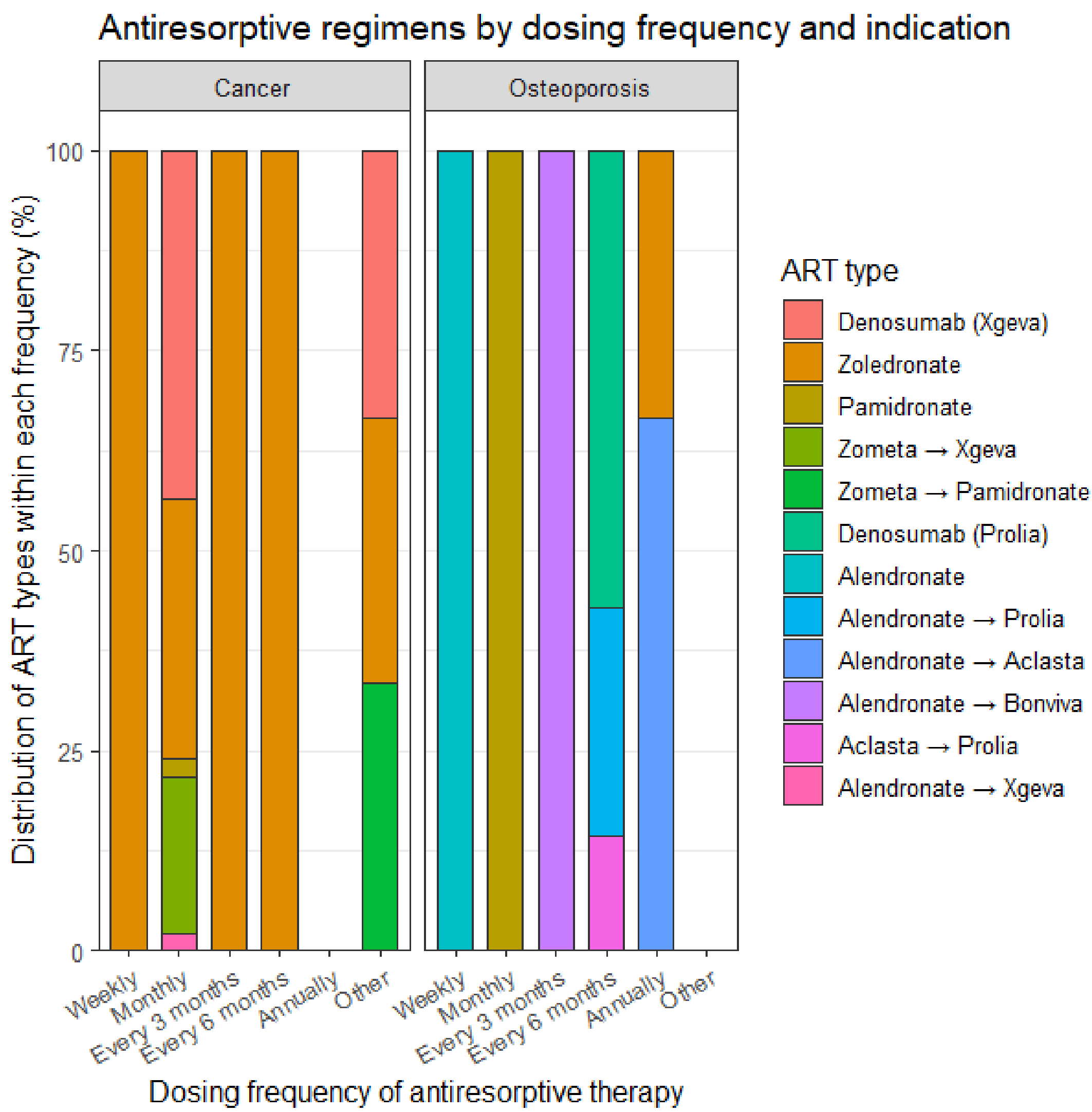

Supplementary Figure S2. Antiresorptive regimens by dosing frequency and indication group. Stacked bar plots show the distribution of antiresorptive drug types within each dosing-frequency category, stratified by indication. Cancer-related MRONJ was dominated by monthly high-intensity regimens, whereas osteoporosis-related MRONJ was dominated by weekly oral alendronate and other lower-frequency regimens.

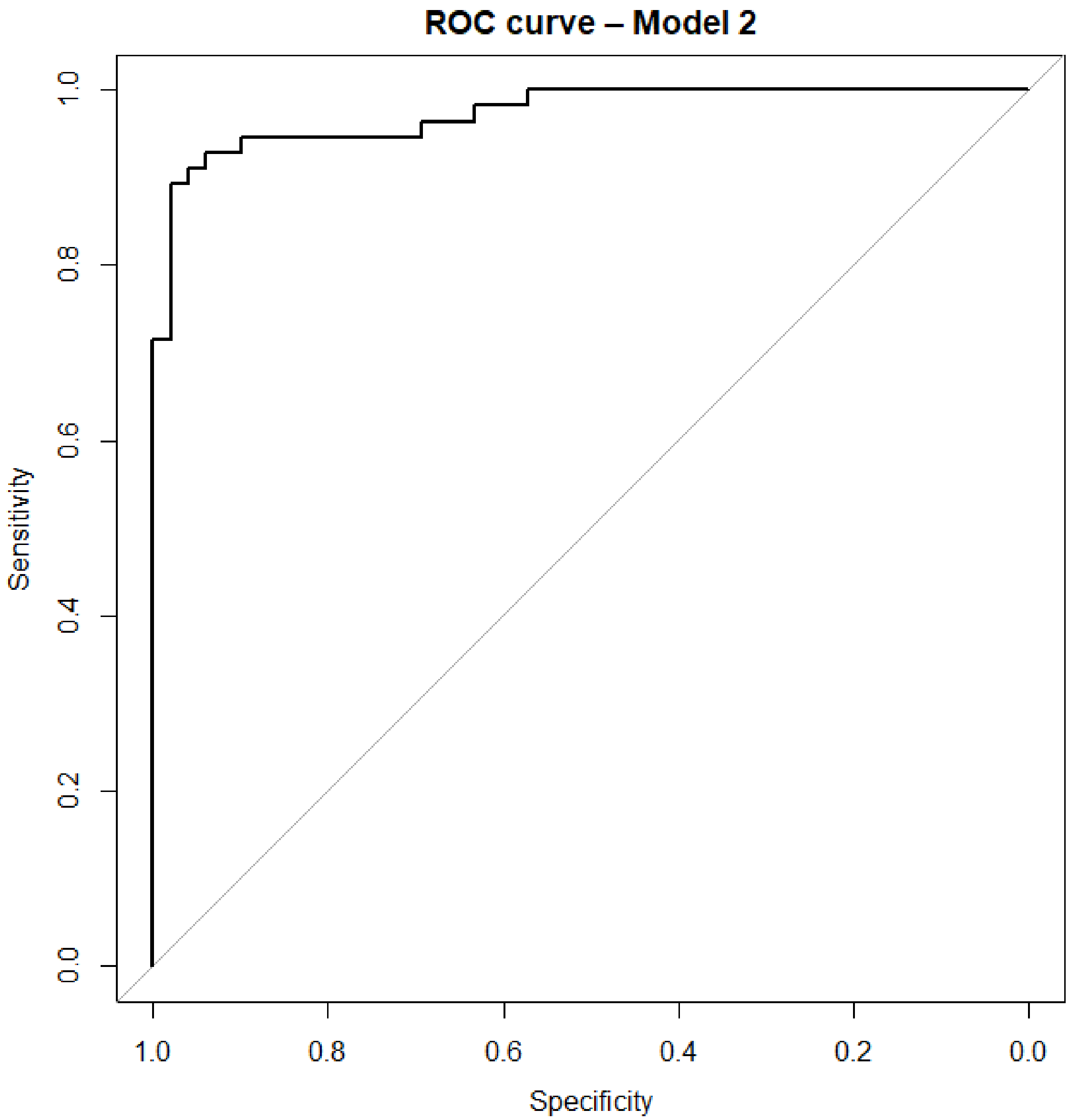

Supplementary Figure S3. Receiver operating characteristic curve for the multivariable indication model. The curve shows the discriminative performance of the logistic regression model distinguishing cancer-related from osteoporosis-related MRONJ among cases.

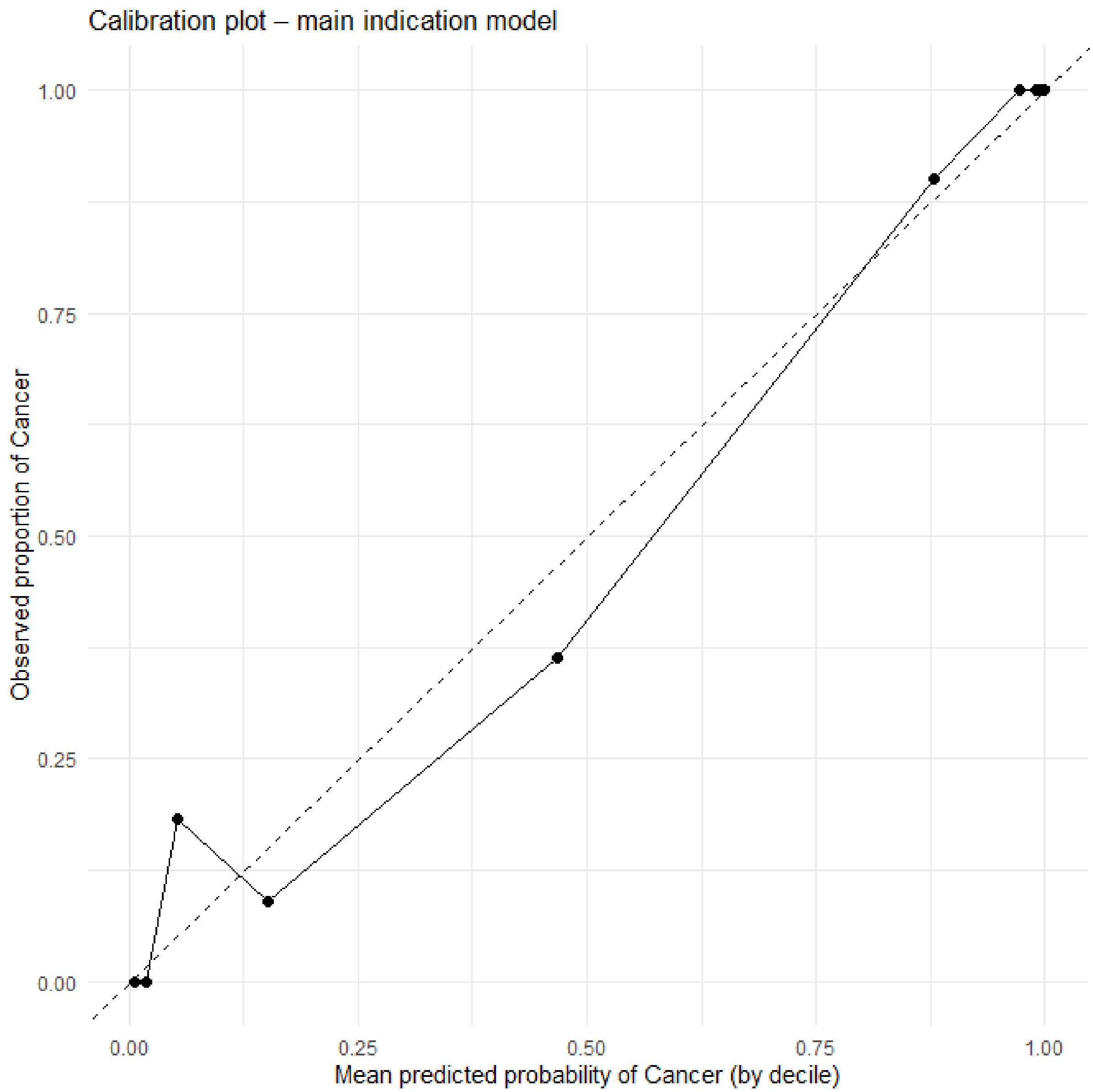

Supplementary Figure S4. Calibration plot for the multivariable indication model. Observed and predicted probabilities of cancer indication are compared across deciles of fitted risk.

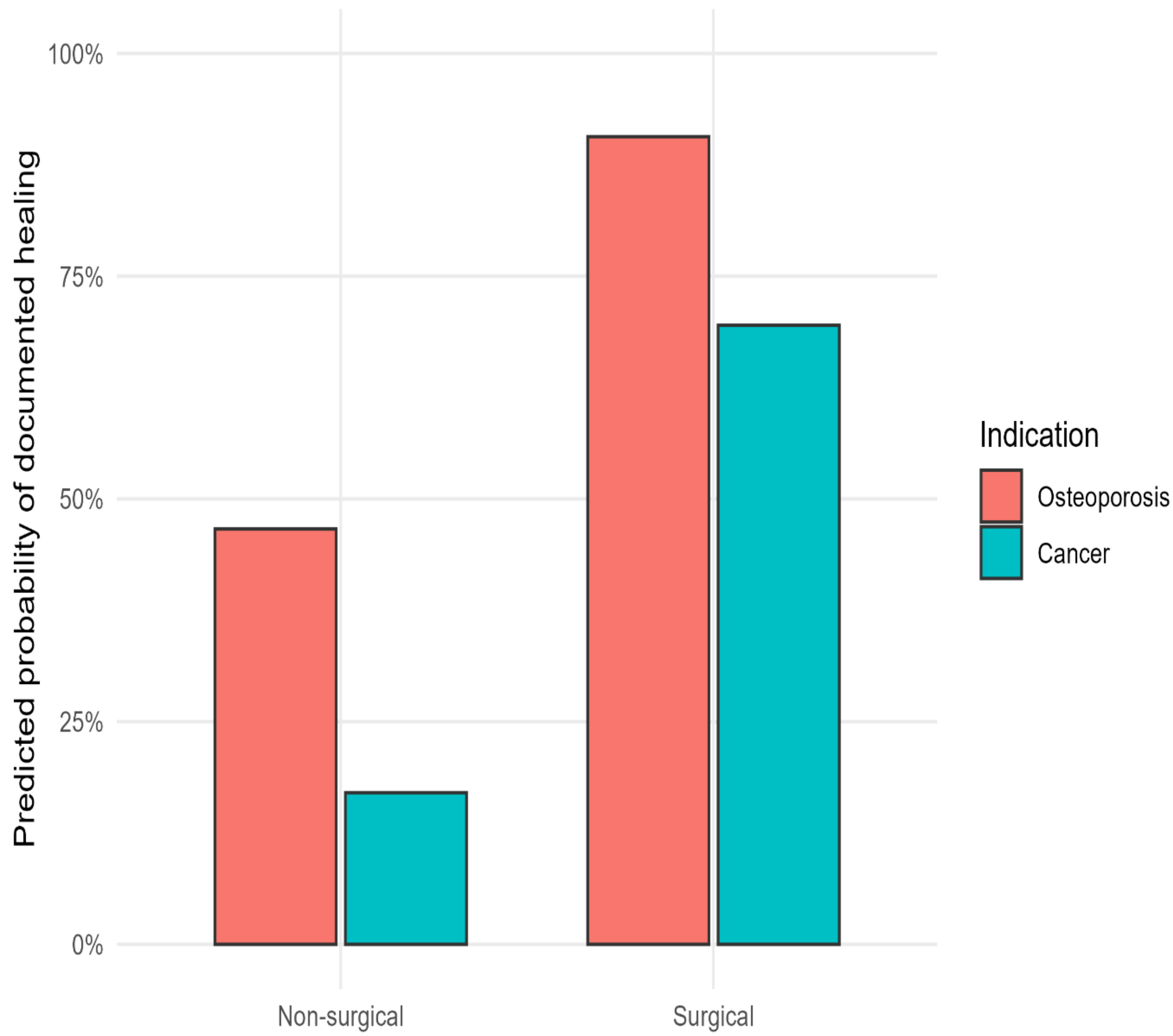

Supplementary Figure S5. Model-based predicted probabilities of documented clinical soft tissue healing by treatment and indication. Predicted probabilities are derived from the primary multivariable logistic regression model at Stage II.

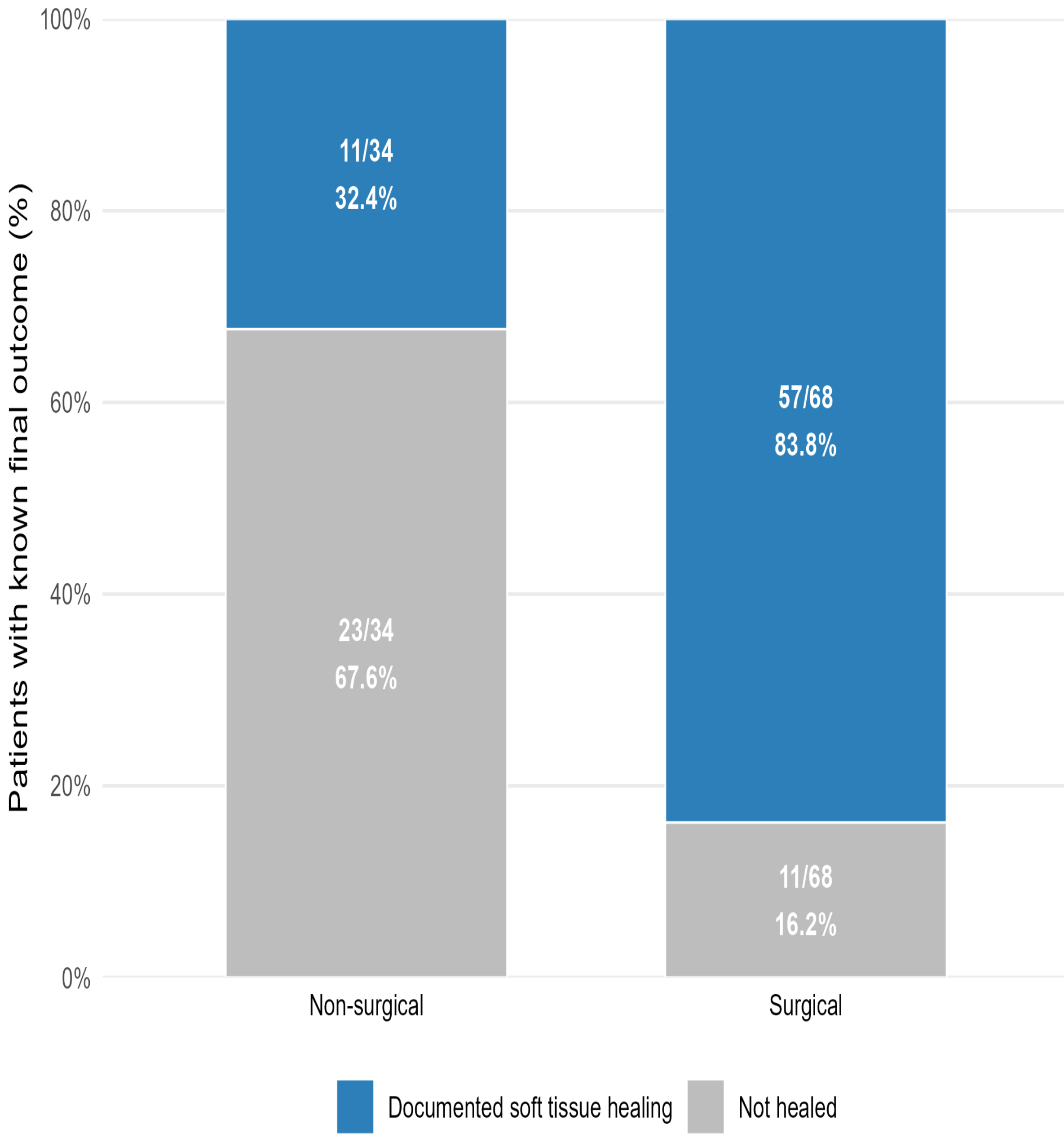

Supplementary Figure S6. Observed documented clinical soft tissue healing proportions by treatment group. Stacked bars show healed and not healed final clinical status among 34 non-surgically managed and 68 surgically treated patients with known final outcome.

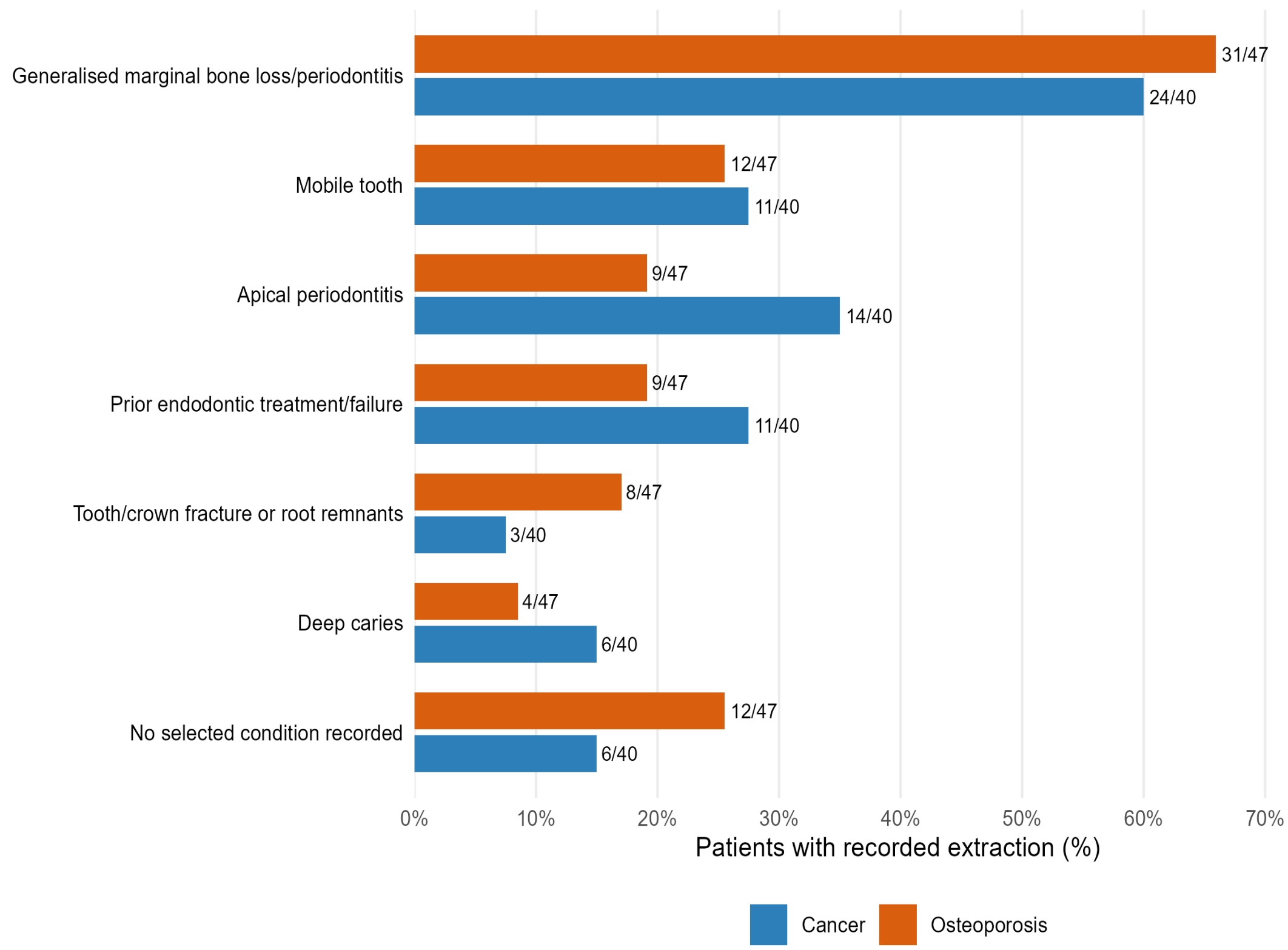

Supplementary Figure S7. Recorded possible reasons or local dental conditions associated with extraction by indication group. Bars show multiple-response proportions among 40 cancer-related and 47 osteoporosis-related cases with recorded extraction. Individual patients could have more than one recorded condition. These data do not establish a verified extraction indication, temporal order, or whether clinically unrecognised MRONJ was already present at extraction.
